# Mapping Resting‐State Brain Functional Specialization to Neurotransmitter Profiles in Autism Spectrum Disorder

**DOI:** 10.1111/cns.70666

**Published:** 2025-11-24

**Authors:** Dafa Shi, Jitian Guan, Guangsong Wang, Shuohua Wu, Caiyu Zhuang, Yumeng Mao, Yanlong Jia, Nannan Zhao, Gen Yan, Renhua Wu

**Affiliations:** ^1^ Department of Radiology The Second Affiliated Hospital of Shantou University Medical College Shantou China; ^2^ Department of Radiology Xiang'an Hospital of Xiamen University, School of Medicine, Xiamen University Xiamen China; ^3^ Department of Radiology Xiangyang Central Hospital, Affiliated Hospital of Hubei University of Arts and Science Xiangyang China; ^4^ Center of Morphological Experiment Medical College of Yanbian University Yanji China; ^5^ Department of Radiology The Second Affiliated Hospital of Xiamen Medical College Xiamen China

**Keywords:** autism spectrum disorder, neuropharmacology, neurotransmitters, regional homogeneity, spatial correlation analysis

## Abstract

**Background:**

Autism spectrum disorder (ASD) is a highly prevalent neurodevelopmental disorder. However, its diagnosis and effective treatment present challenges. Understanding neurotransmitter impairments may offer new perspectives into the mechanisms underlying ASD and the potential therapeutic targets for this condition. This study aimed to investigate the spatial associations of ASD‐related brain activity patterns and multiple specific neurotransmitter distributions to identify abnormal neurotransmitter alterations in patients with ASD, and to assess how these spatial associations relate to clinical features.

**Methods:**

We included 44 patients with ASD and 132 typically developing controls (TDCs) and compared the regional homogeneity (ReHo) differences between the two groups. Associations between the spatial patterns of ReHo alterations and specific neurotransmitter receptor/transporter densities in patients with ASD were evaluated, and the correlations of these associations with the clinical characteristics were analyzed.

**Results:**

In comparison with TDCs, patients with ASD exhibited specific brain activity abnormalities in the visuomotor network, cerebro‐cerebellar circuits, angular gyrus, and limbic areas. These atypical brain activity patterns were significantly co‐localized with the serotonergic, glutamatergic, GABAergic, dopaminergic, noradrenergic, cholinergic, and cannabinoid neurotransmitter systems in patients with ASD, and the results showed good reproducibility between different neurotransmitter maps. Additionally, the awareness score in the Social Responsiveness Scale (*ρ* = −0.475, *p* = 0.009) and the social score in the Autism Diagnostic Observation Schedule (*ρ* = −0.415, *p* = 0.049) exhibited negative correlations with the strength of ReHo co‐localization of serotonin 5‐hydroxytryptamine receptor subtype 2a.

**Conclusions:**

This is the first systematic analysis of multiple neurotransmitter systems to show abnormalities in these systems in patients with ASD. These results will enhance the existing understanding of the mechanisms underlying ASD and may provide the foundation for identifying therapeutic targets.

## Graphical abstract

Our multimodal neuroimaging analysis integrating MRI and PET/SPECT data revealed robust and consistent abnormalities across multiple neurotransmitter systems in patients with ASD, including serotonergic, glutamatergic, GABAergic, dopaminergic, noradrenergic, acetylcholinergic, and cannabinoid systems.

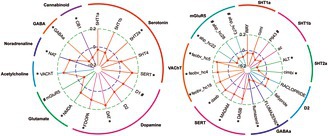

## 1 Background

Autism spectrum disorder (ASD) is a highly prevalent neurodevelopmental disorder with a rapidly increasing incidence, affecting 1%–2% of the global population [1, 2]. Although ASD exhibits common core features, including restricted and repetitive behaviors and impaired social communication and interaction and/or interests [3, 4], its clinical manifestations are markedly heterogeneous [2, 5, 6]. However, the clinical diagnosis of ASD relies primarily on behavioral observations, and no effective biomarkers for the diagnosis of ASD are available at present. Furthermore, despite extensive efforts to develop pharmacological treatments for ASD, no approved medications that target its core symptoms are currently available [7, 8, 9]. The neurobiological basis of ASD is not well understood [3, 10], and deeper insights into its mechanisms of action are required to achieve early diagnosis and effective treatment.

Neuroimaging techniques, including structural and functional magnetic resonance imaging (MRI), positron emission tomography (PET), and single‐positron emission computed tomography (SPECT), allow noninvasive assessment of brain structure, function, metabolism, and neurochemical changes [4, 11, 12, 13, 14, 15]. They offer the potential to better understand the underlying neurophysiological mechanisms of ASD [4, 8, 11]. Resting‐state functional MRI (rs‐fMRI) is commonly used to characterize brain activity [5, 8, 14, 15]. Regional homogeneity (ReHo) is a data‐driven measurement of local spontaneous neural activity based on rs‐fMRI data. It reflects regional activity synchronizations between a voxel and its nearest neighboring voxels [16, 17]. Previous studies [16, 17, 18] have demonstrated specific ReHo abnormalities in ASD, indicating the utility of ReHo analyses in ASD. Additionally, in comparison with other commonly used rs‐fMRI measures, ReHo correlates more closely with regional cerebral glucose metabolism [19, 20]. Consequently, ReHo has been used to quantitatively assess ASD‐related brain activity patterns and analyze their spatial association with neurotransmitters. PET‐ and SPECT‐based molecular imaging studies have shown that ASD involves the dysfunction of several neurotransmitter systems, including the serotoninergic [21], dopaminergic [22, 23], GABAergic [24, 25], noradrenergic [23] and glutamatergic [8] systems. Therefore, drugs targeting these neurotransmitter systems may serve as potential therapeutic candidates for ASD [8, 26, 27, 28, 29].

Advances in neuroimaging have facilitated our understanding of the neurobiological mechanisms of ASD, its clinical diagnosis and evaluation, and the development of biomarkers and targeted therapeutics. However, most studies employed analyses at the voxel or regional level, exhibiting low test–retest reliability [30, 31]. Furthermore, these analyses did not elucidate the underlying neurophysiological mechanisms of the diseases [30]. In contrast, recent reports [30, 31, 32, 33, 34] have shown that the overall spatial activity patterns yielded more reliable results. Moreover, spatial correlations between underlying biological and imaging alterations can better reveal the mechanisms underlying neurobiological diseases. Hansen et al. [35] revealed spatial correlations between the distribution of neurotransmitter receptors/transporters and cortical abnormality patterns in 13 disorders. Other studies [32, 36] have also revealed complex mapping relationships between the effects of drugs on brain function and multiple neurotransmitter systems, providing valuable insights into the effects of pharmacological interventions on brain function and potentially identifying therapeutic targets. Dukart et al. [30] developed the JuSpace toolbox to analyze the spatial associations of PET/SPECT‐derived maps, including multiple neurotransmitter systems, with MRI‐derived or other imaging modalities. This approach has been applied to study Parkinson disease [30, 33, 37], multiple sclerosis [34], and prodromal frontotemporal dementia [38]. Our previous study [33] showed that this approach is highly reproducible for assessing disease‐related neurotransmitter system abnormalities and is useful for identifying potential therapeutic targets. In summary, investigating abnormalities in the overall spatial activity patterns in ASD and their spatial associations with neurotransmitters holds promise for providing new insights into the neuropathological mechanisms of ASD and for identifying potential targets for therapeutic interventions.

The associations among brain activity, neurotransmitter receptor/transporter distribution, and the clinical characteristics of ASD remain poorly understood. In this study, we explored the spatial associations of ASD‐related brain activity patterns identified using ReHo measurements with neurotransmitter receptor/transporter distributions to assess their relationships with clinical features.

## 2 Materials and Methods

### 2.1 Participants

**FIGURE 1:**
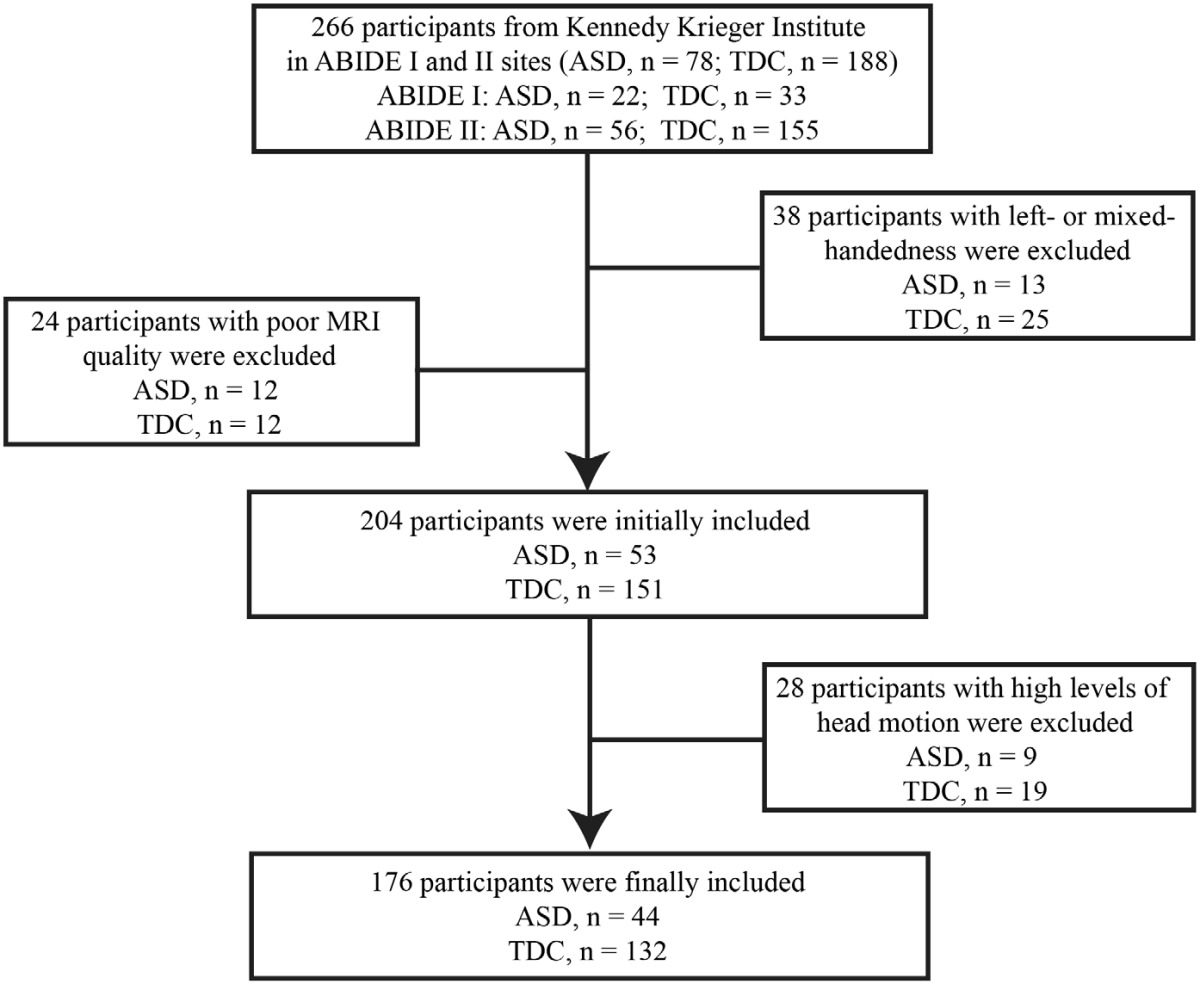
Flow diagram of participants' selection process. ABIDE, autism brain imaging data exchange; ASD, autism spectrum disorder; TDC, typically developing control.

Original MRI and demographic data of the participants were downloaded from the open‐access Autism Brain Imaging Data Exchange (ABIDE) repository (ABIDE I & II sites, https://fcon_1000.projects.nitrc.org/indi/abide) [39, 40]; specifically, we downloaded the data from the Kennedy Krieger Institute (KKI), which includes 78 patients with ASD and 188 typically developing controls (TDCs; ASD, *n* = 78; TDC, *n* = 188). The use of single‐site data can reduce potential center‐ and scanner‐related variability, and data from the early stage is more effective for ASD diagnosis. The KKI dataset had the largest sample within the age range of 8–13 years. All ASD diagnoses were confirmed using the Autism Diagnostic Interview‐Revised and/or the Autism Diagnostic Observation Schedule‐Generic (ADOS‐G) module 3 or the Autism Diagnostic Observation Schedule, Second Edition (ADOS‐2) module 3. The more details on diagnosis of ASD, along with the inclusion and exclusion criteria for patients with ASD and TDCs, are available at https://fcon_1000.projects.nitrc.org/indi/abide/abide_I.html and https://fcon_1000.projects.nitrc.org/indi/abide/abide_II.html. Participants were excluded on the basis of the following criteria: (1) left‐ or mixed‐handedness (ASD, *n* = 13; TDC, *n* = 25), (2) poor MRI image quality (ASD, *n* = 12; TDC, *n* = 12), or (3) high levels of head motion (maximum head motion greater than 3 mm or 3°; ASD, *n* = 9; TDC, *n* = 19). Lastly, 44 patients with ASD and 132 TDCs were included in our study (Figure 1). Ethical approval was obtained from the Institutional Review Board of KKI and complied with the Declaration of Helsinki, and written informed consent was acquired from all participants or their parent/guardian.

### 2.2 Clinical Assessment

Clinical assessment to determine the intelligence quotient (IQ) and assessments based on ADOS, Social Responsiveness Scale (SRS), and Autism Diagnostic Interview‐Revised (ADI‐R) were performed. The detailed demographic and clinical information of the participants is summarized in Table 1.

**TABLE 1:** Demographic and clinical data.

	ASD (*N* = 44)	TDC (*N* = 132)	Statistics	*p‐value*
Age	10.40 ± 1.52	10.34 ± 1.18	*t* = 0.28	0.78
Sex (M/F)	33/11	80/52	*χ* ^2^ = 2.98	0.09
FIQ (N_ASD_ = 43)	102.33 ± 15.78	114.42 ± 10.35	*t* = −4.71	< 0.01
VIQ (N_ASD_ = 33; N_TDC_ = 107)	111.42 ± 14.74	117.84 ± 12.05	*t* = −2.28	0.03
PIQ (N_ASD_ = 33; N_TDC_ = 107)	105.36 ± 14.06	110.53 ± 12.81	*t* = −1.98	0.05
ADI_R_Social (N_ASD_ = 43)	19.21 ± 5.91	—	—	—
ADI_R_Verbal (N_ASD_ = 43)	15.05 ± 5.01	—	—	—
ADI_R_Noverbal (N_ASD_ = 32)	8.00 ± 3.39	—	—	—
ADI_R_RRB (N_ASD_ = 43)	6.00(4.00–7.00)	—	—	—
ADOS_Total (N_ASD_ = 26)	13.88 ± 3.32	—	—	—
ADOS_ Communication (N_ASD_ = 26)	3.00(3.00–4.00)	—	—	—
ADOS_ Social (N_ASD_ = 26)	8.00(6.75–9.00)	—	—	—
ADOS_STEREO (N_ASD_ = 26)	2.93 ± 1.54	—	—	—
SRS_Total (N_ASD_ = 32; N_TDC_ = 104)	69.19 ± 27.82	15.91 ± 10.43	*t* = 15.98	< 0.01
SRS_Awareness (N_ASD_ = 32; N_TDC_ = 104)	13.34 ± 3.70	4.12 ± 2.61	*t* = 13.12	< 0.01
SRS_Cognition (N_ASD_ = 32; N_TDC_ = 104)	16.62 ± 6.23	2.00(1.00–4.00)	*Z* = −8.26	< 0.01
SRS_Communication (N_ASD_ = 32; N_TDC_ = 104)	32.59 ± 9.49	3.00(1.00–6.00)	*Z* = −8.54	< 0.01
SRS_Motivation (N_ASD_ = 32; N_TDC_ = 104)	14.66 ± 6.35	3.00(1.00–5.00)	*Z* = −7.73	< 0.01
SRS_Mannerisms (N_ASD_ = 32; N_TDC_ = 104)	18.97 ± 6.95	1.00(1.00–2.00)	*Z* = −8.64	< 0.01
Coil (8/32 channel)	35/9	105/27	*χ* ^2^ = 0	1.00

*Note:* Data are presented as mean ± SD for normally distributed data or median (interquartile range) for non‐normally distributed data. Comparisons were performed using the two‐sample *t*‐test, chi‐square test, and Mann–Whitney U test.

Abbreviations: ADI‐R, autism diagnostic interview‐revised; ADOS, autism diagnostic observation schedule; ASD, autism spectrum disorder; F, female; FIQ, full‐scale IQ; IQ, intelligence quotient; M, male; PIQ, performance IQ; RRB, restricted and repetitive behaviors; SRS, Social Responsiveness Scale; STEREO, stereotyped behaviors; TDC, typically developing control; VIQ, verbal IQ.

### 2.3 Neuroimaging Acquisition and Preprocessing and ReHo Calculation

Rs‐fMRI and high‐resolution 3D T1‐weighted structural MRI data were acquired using two 3 Tesla Philips scanners (Achieva; Philips Healthcare, Best, Netherlands) with an eight‐channel or 32‐channel phased‐array head coil. ABIDE I data were acquired using an eight‐channel head coil, whereas ABIDE II data were acquired using an eight‐ or 32‐channel head coil. For the data collected using the two scanners, rs‐fMRI was performed using the same scanning parameters, and the 3D T1‐weighted anatomical data were obtained with similar scanning parameters. Rs‐fMRI images were acquired using echo‐planar imaging pulse sequences: 156 volumes, slices = 47, repetition time/echo time = 2500/30 ms, field of view = 256 mm × 256 mm, slice thickness = 3.0 mm, no gap, matrix = 96 × 96, voxel size = 2.67 × 2.67 × 3.00 mm^3^, and flip angle = 75°. For structural data, the voxel sizes of the eight‐ and 32‐channel scanners were 1.0 × 1.0 × 1.0 mm^3^ and 0.95 × 0.96 × 1.0 mm^3^, respectively. The percentages of data collected using the two scanners in patients with ASD and TDCs matched perfectly (*χ* ^2^ = 0, *p* = 1.00; Table 1). Scan parameters are listed more specifically in Supporting Information S1.

ReHo analysis was conducted using the Data Processing and Analysis for (Resting‐State) Brain Imaging (DPABI) toolbox [41]. The preprocessing steps were as follows: removal of the first eight volumes (20 s), slice timing correction, image realignment (participants were excluded if they had a maximum translation greater than 3.0 mm or a maximum rotation greater than 3.0°), spatial normalization and resampling (3 × 3 × 3 mm^3^), regression of nuisance variables (including white matter, cerebrospinal fluid and global signal, and 24 head motion parameters) and linear drift, and bandpass filtering (0.01–0.08 Hz). The specific preprocessing procedures for ReHo are detailed in Supporting Information S1 and in our previous study [42]. Individual ReHo mapping was performed using a voxel‐wise approach by calculating Kendall's coefficient of concordance for each voxel's time series with its 26 nearest‐neighboring voxels. This measures local BOLD signal synchrony by assessing the rank concordance of time series within functionally homogeneous regions. Next, the ReHo value of each voxel was transformed into a z‐score for standardization to minimize the influence of individual variability [42, 43]. Finally, the ReHo maps were spatially smoothed using a 4‐mm full‐width at half‐maximum isotropic Gaussian kernel.

### 2.4 Data Harmonization

The ComBat harmonization technique [44, 45] was used after ReHo spatial smoothing and before downstream statistical analyses to reduce potential biases and non‐biological variability induced by differences in MRI scanners and to maximize statistical power. ComBat harmonization employs a multivariate linear mixed‐effects regression and empirical Bayes modeling method for data harmonization and has been successfully applied to diffusion tensor imaging [45], rs‐fMRI [5, 46], and structural imaging [47]. Age, sex, and group (ASD or TDC) were included as biological variables of interest in the harmonization process [5, 46]. We performed nonparametric adjustments using the ComBat function (i.e., setting the ComBat function parameter to 0; https://github.com/Jfortin1/ComBatHarmonization). In this setting, ComBat determines the transformation of each voxel ReHo value separately [48].

### 2.5 Selection of Neurotransmitter PET/SPECT Maps

Recent studies [8, 26, 27, 28, 29] have systematically reviewed the roles of the serotonergic, dopaminergic, GABAergic, glutamatergic, and cholinergic neurotransmitter systems in the diagnosis and targeted therapy of ASD. Emerging evidence has indicated that the noradrenergic system also plays an important role in ASD [23, 49]. Additionally, while some studies [50, 51] have suggested that drugs targeting the endocannabinoid system may have therapeutic potential for ASD, another study [52] failed to confirm their efficacy; thus, their clinical value requires further validation [53, 54]. On the basis of these findings, this study aimed to explore the spatial association between abnormal spatial activity patterns and the distributions of these neurotransmitter systems. To this end, we selected the 15 neurotransmitter PET/SPECT maps from the JuSpace toolbox [30], including (1) serotonergic: 5‐hydroxytryptamine (5HT) receptor subtypes 1a, 1b, 2a, and 4 (5HT1a, 5HT1b, 5HT2a, and 5HT4), and serotonin transporter (SERT); (2) glutamatergic: metabotropic glutamate receptor 5 (mGluR5), and *N*‐methyl‐ d‐aspartic acid receptor (NMDA); (3) dopaminergic: dopamine D1 and D2 receptors (D1 and D2), ^18^F fluorodopa (FDOPA), and dopamine transporter (DAT); (4) noradrenergic: noradrenaline transporter (NAT); (5) cholinergic: vesicular acetylcholine transporter (VAChT); (6) gamma‐aminobutyric acid (GABA) type a receptor (GABAa); and (7) cannabinoid 1 receptor (CB1). The details are shown in Supporting Informations S2, Table S1 and Figure S1.

### 2.6 Validation Analysis

In the JuSpace toolbox, multiple neurotransmitter receptor/transporter maps are available with multiple maps, including those for 5HT1a, 5HT1b, 5HT2a, D2, GABAa, SERT, VAChT, and mGluR5 (Supporting Information S2, Table S2). We used multiple maps of the same neurotransmitter receptor/transporter to validate the robustness and reproducibility of the results.

### 2.7 Statistical Analysis

Demographic and clinical scores were compared using the two‐sample *t*‐test, Mann–Whitney U test, and chi‐square test, as appropriate. Continuous variables were assessed for normality and homogeneity of variance using the Shapiro–Wilk and Levene's tests. Normally distributed data were analyzed using independent two‐sample *t*‐tests. Non‐normally distributed continuous variables were analyzed using the nonparametric Mann–Whitney U tests. Categorical variables were compared using the chi‐square tests.

**FIGURE 2:**
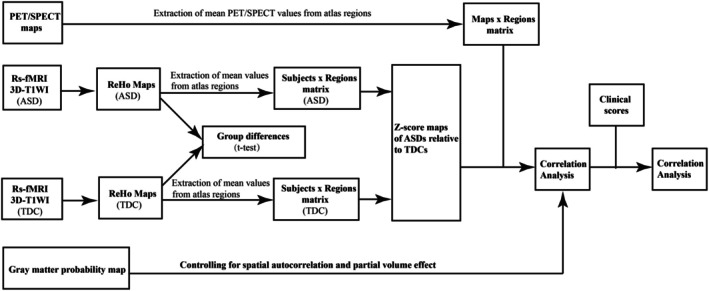
Flowchart of the statistical analysis pipeline. ASD, autism spectrum disorder; TDC, typically developing control; PET, positron emission tomography; SPECT, single‐positron emission computed tomography.

A flowchart of the statistical analysis pipeline used in this study is shown in Figure 2.

We performed a two‐sample *t*‐test using the DAPBI toolbox to assess group differences in ReHo between TDCs and patients with ASD, with age, sex, and head coil (8‐channel or 32‐channel) used as covariates. Gaussian random field (GRF) theory correction was applied for multiple comparisons (*p* < 0.001 at the voxel level and *p* < 0.05 at the cluster level).

The JuSpace toolbox (version 1.5, https://github.com/juryxy/JuSpace) [30] was used to evaluate the spatial associations between the spatial patterns of ReHo alterations in patients with ASD relative to TDCs and the specific neurotransmitter receptor/transporter distribution maps of PET/SPECT. Covariates, such as age, sex, and head coil, were adjusted by regression before the spatial correlation analysis between the spatial patterns of ReHo alterations and specific neurotransmitter receptor/transporter density maps [33, 38]. To quantify ReHo alterations in patients with ASD relative to TDCs, we implemented the following computational procedure: First, the group‐level mean (Mean_Reho_TDC_) and standard deviation (Std_ReHo_TDC_) of ReHo values were calculated for the TDC cohort. Subsequently, the ReHo map of each patient with ASD was converted to a *z*‐score by subtracting Mean_Reho_TDC_ and dividing by Std_ReHo_TDC_. This process generated an individualized ReHo alteration map representing the spatial pattern and magnitude of deviation in each patient with ASD relative to the TDC population. All analyses were performed using the age‐, sex‐, and head coil‐adjusted *z*‐transformed individual ReHo maps. Then, Fisher's *z*‐transformed Spearman's correlation coefficients were calculated between *z*‐transformed individual ReHo maps of patients with ASD relative to TDCs and the selected neurotransmitter receptor/transporter maps (15 and 19 neurotransmitter maps for the primary and validation analyses, respectively). Calculations were performed using the JuSpace toolbox [30] with computing option 5 and the Brainnetome 246 atlas [55] (Supporting Informations S3, Table S3 and Figure S2). The gray matter probability map was used to adjust for spatial autocorrelation and partial‐volume effects. To assess the statistical significance of the observed correlation coefficients, exact permutation‐based *p*‐values were calculated using 10,000 permutations to determine whether the observed correlation coefficients were statistically different from zero using one‐sample *t*‐tests. The false discovery rate (FDR) was applied to correct for multiple comparisons (the number of neurotransmitter maps: 15 and 19 for the primary and validation analyses, respectively) (Figure 2). Further details are provided in previous studies [30, 33, 34] and Supporting Informations S4.

Next, using Spearman's partial correlation analysis, Fisher's z‐transformed Spearman's correlation coefficients (i.e., ReHo‐neurotransmitter spatial associations) were examined in conjunction with the clinical assessment scores of patients with ASD, including the ADOS, ADI‐R and SRS total and subscale scores. Age, sex, and head coil were included as covariates. In the above correlation analysis, we included only significant ReHo‐neurotransmitter receptor/transporter correlation coefficients.

Unless otherwise specified, all tests were two‐sided, with the significance level set at *p* < 0.05.

## 3 Results

### 3.1 Participant Characteristics

Table 1 shows the demographic and clinical characteristics of all the participants. No significant differences in age, sex, or head coil were found between patients with ASD and TDCs (all *p* > 0.05). However, patients with ASD exhibited lower full‐scale and verbal IQ (both *p* < 0.05) and higher SRS total and subscale scores than TDCs (all *p* < 0.01) (Table 1).

### 3.2 ReHo Alterations in Patients With ASD

**FIGURE 3:**
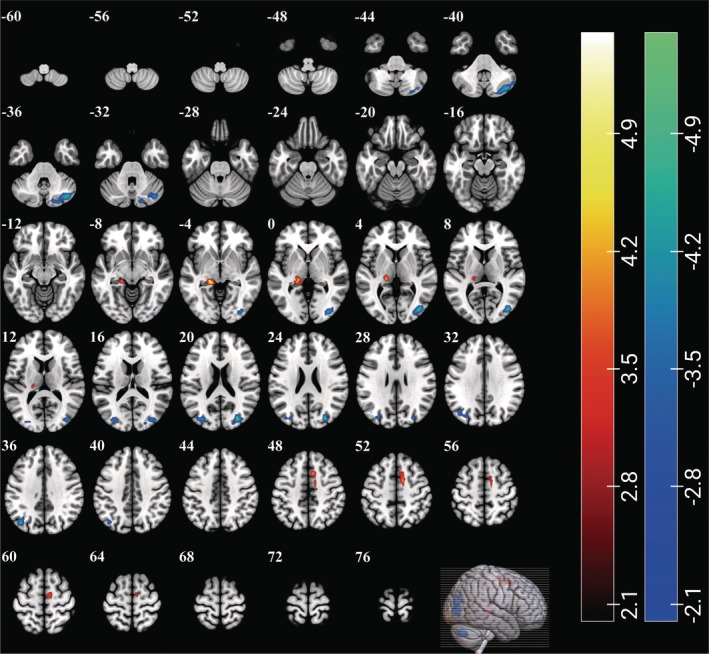
ReHo changes in patients with ASD. Brain regions showing significant differences were the bilateral middle occipital gyrus, right cerebellum crus I and II, supplementary motor area, left angular gyrus, thalamus, parahippocampal gyrus, and hippocampus (GRF‐corrected, *p* voxel < 0.001, *p* cluster < 0.05, adjusted for age, sex, and head coil). The colorbars represent the *t*‐values: Positive values indicate higher ReHo values in patients with ASD than in TDCs (patients>controls), while negative values indicate the opposite (patients<controls).

**FIGURE 4:**
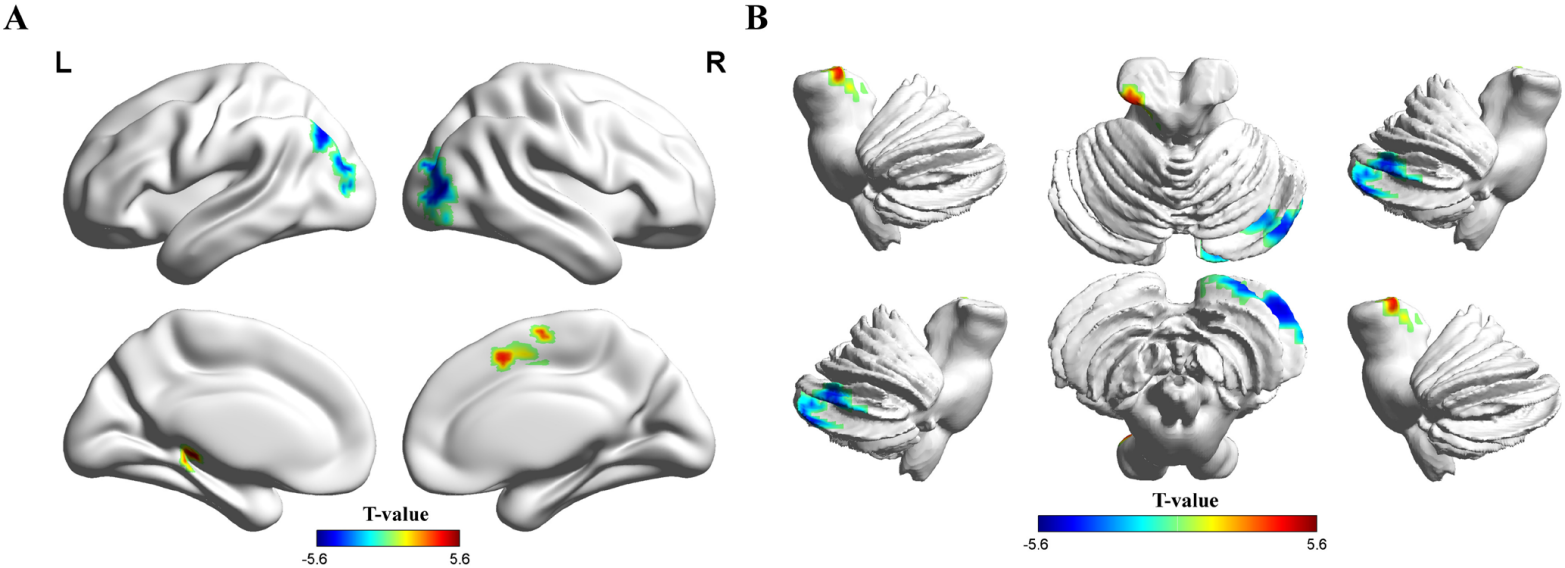
Differences in ReHo between patients with ASD and TDCs. Brain regions showing significant differences were the bilateral middle occipital gyrus, right cerebellum crus I and II, supplementary motor area, and left angular gyrus, thalamus, parahippocampal gyrus, and hippocampus. The colorbars represent the *t*‐values: Warmer hues (positive values) indicate higher ReHo values in patients with ASD than in TDCs (patients>controls), while cooler hues (negative values) indicate the opposite (patients<controls).

ReHo values in the left thalamus, hippocampus, parahippocampal gyrus (PHG), and right supplementary motor area (SMA) were significantly higher in patients with ASD than in TDCs. Furthermore, the ReHo values in the bilateral middle occipital gyrus (MOG), right cerebellum crus I and II, and left angular gyrus were lower in patients with ASD than in TDCs (GRF‐corrected, *p* < 0.001 at the voxel level and *p* < 0.05 at the cluster level, adjusted for age, sex, and head coil) (Figures 3 and 4, and Supporting Informations S5, Table S4).

### 3.3 Spatial Associations Between ReHo Alterations and Neurotransmitter Receptor/Transporter Density Maps

**FIGURE 5:**
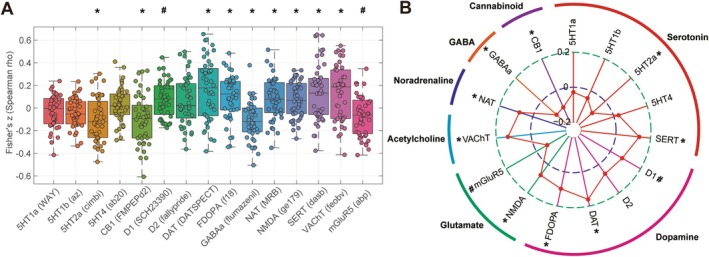
Findings of the spatial correlation analysis between ReHo alterations and neurotransmitter receptor/transporter density distributions. Box plot with scatter (A) and radar plot (B) showed that relative to TDCs, ReHo alterations in patients with ASD were significantly associated with the spatial distribution of serotonergic, dopaminergic, noradrenergic, GABAergic, and cannabinoid neurotransmitter maps. For details of the neurotransmitter receptor/transporter map information and abbreviations, see Supporting Informations S2, Table S1, and Figure S1. *p < 0.05, FDR‐corrected; ^#^ *p* < 0.05, uncorrected.

Significant associations were observed between ReHo alterations in patients with ASD in comparison with TDCs and the spatial distribution of diverse neurotransmitter systems. Specifically, we found associations with serotonergic (5HT2a, *ρ* = −0.0998, *p* = 0.0122; SERT, *ρ* = 0.1352, *p* = 0.0124), glutamatergic (NMDA, *ρ* = 0.0752, *p* = 0.0226), GABAergic (GABAa, *ρ* = −0.1141, *p* = 0.0016), dopaminergic (FDOPA, *ρ* = 0.0993, *p* = 0.0221; DAT, *ρ* = 0.1613, *p* = 0.0075), cholinergic (VAChT, *ρ* = 0.1330, *p* = 0.0086), noradrenergic (NAT, *ρ* = 0.0806, *p* = 0.0050), and cannabinoid (CB1, *ρ* = −0.1027, *p* = 0.0153) neurotransmitter receptor/transporter maps (all *p* < 0.05, FDR‐corrected). Additionally, mGluR5 (*ρ* = −0.0714, *p* = 0.0371) and D1 (*ρ* = 0.0787, *p* = 0.0369) receptor maps showed a significant trend but did not meet the threshold for significance after FDR correction (*p*_FDR‐corrected_ = 0.0226) (Figure 5 and Table 2).

**TABLE 2:** Results of spatial correlation analyses between ReHo alterations and neurotransmitter receptor/transporter density maps.

Receptor/transporter	Neurotransmitter	Spearman *ρ*	Exact *p‐value*	FDR‐corrected *p‐value*
5HT1a	Serotonin	– 0.0285	0.2919	0.3127
5HT1b	Serotonin	– 0.0455	0.0593	0.0741
5HT2a	Serotonin	– 0.0998	0.0122^#^	0.0310*
5HT4	Serotonin	0.0278	0.3848	0.3848
CB1	Cannabinoid	– 0.1027	0.0153^#^	0.0328*
D1	Dopamine	0.0787	0.0369^#^	0.0540
D2	Dopamine	0.0647	0.1619	0.1868
DAT	Dopamine	0.1613	0.0075^#^	0.0310*
FDOPA	Dopamine	0.0993	0.0221^#^	0.0377*
GABAa	GABA	– 0.1141	0.0016^#^	0.0240*
NAT	Noradrenaline	0.0806	0.0050^#^	0.0310*
NMDA	Glutamate	0.0752	0.0226^#^	0.0377*
SERT	Serotonin	0.1352	0.0124^#^	0.0310*
VAChT	Acetylcholine	0.1330	0.0086^#^	0.0310*
mGluR5	Glutamate	– 0.0714	0.0371^#^	0.0540

*Note:* For details of the neurotransmitter receptor/transporter map information and abbreviations, see Supporting Information S2, Table S1, and Figure S1. ^#^ *p* < 0.05, uncorrected; *p < 0.05, FDR‐corrected.

### 3.4 Validation Analysis

**FIGURE 6:**
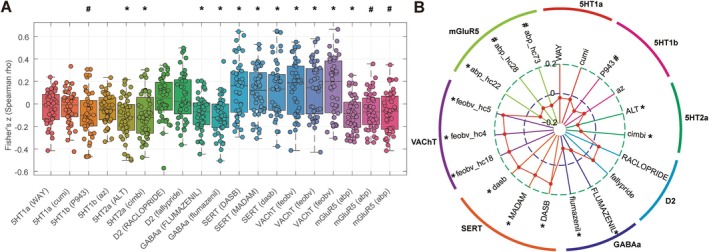
Findings of the spatial correlation analysis between ReHo alterations and different maps of the same neurotransmitter receptor/transporter map. Box plot with scatter (A) and radar plot (B) showed that the spatial correlation analysis had good reproducibility between different maps of the same neurotransmitter receptor/transporter map. For details of the neurotransmitter receptor/transporter map information and abbreviations, see Supporting Informations S2, Table S1 and Figure S1. *p < 0.05, FDR‐corrected; ^#^ *p* < 0.05, uncorrected.

To validate the robustness and reproducibility of our results, we used different maps of the same neurotransmitter receptor/transporter to replicate the spatial correlation analysis described above. We observed consistent results for 5HT1a, 5HT2a, D2, GABAa, SERT, and VAChT. Specifically, significant spatial correlations were observed between ReHo alterations and 5HT2a, GABAa, SERT, and VAChT expressions in patients with ASD relative to TDCs (all *p* < 0.05, FDR‐corrected), whereas no significant correlations were observed between 5HT1a and D2 (all *p* > 0.05). Similar results were observed for 5HT1b and mGluR5 on different maps. Further details are provided in Figure 6 and Table 3.

**TABLE 3:** Results of spatial correlation analysis between ReHo alterations and different maps of the same neurotransmitter receptor/transporter map.

Receptor/transporter	Map	Spearman ρ	Exact *p*‐value	FDR‐corrected *p*‐value
5HT1a	5HT1a_WAY_HC36	−0.0285	0.2883	0.3043
	5HT1a_cumi_hc8_beliveau	−0.0277	0.3240	0.3240
5HT1b	5HT1b_P943_HC22	−0.0851	0.0365^#^	0.0571
	5HT1b_az_hc36_beliveau	−0.0455	0.0631	0.0799
5HT2a	5HT2a_ALT_HC19	−0.1179	0.0018^#^	0.0199*
	5HT2a_cimbi_hc29_beliveau	−0.0998	0.0126^#^	0.0266*
D2	D2_RACLOPRIDE_c11	0.0456	0.2302	0.2573
	D2_fallypride_hc49_jaworska	0.0647	0.1643	0.1951
GABAa	GABAa_FLUMAZENIL_c11	−0.0840	0.0224^#^	0.0387*
	GABAa_flumazenil_hc16_norgaard	−0.1141	0.0021^#^	0.0199*
SERT	SERT_DASB_HC30	0.1309	0.0153^#^	0.0291*
	SERT_MADAM_c11	0.1087	0.0100^#^	0.0245*
	SERT_dasb_hc100_beliveau	0.1352	0.0103^#^	0.0245*
VAChT	VAChT_feobv_hc18_aghourian	0.1330	0.0100^#^	0.0245*
	VAChT_feobv_hc4_tuominen	0.1331	0.0097^#^	0.0245*
	VAChT_feobv_hc5_bedard	0.1545	0.0040^#^	0.0245*
mGluR5	mGluR5_abp_hc22_rosaneto	−0.0864	0.0057^#^	0.0245*
	mGluR5_abp_hc28_dubois	−0.0609	0.0492^#^	0.0668
	mGluR5_abp_hc73_smart	−0.0714	0.0391^#^	0.0571

*Note:* For details of the neurotransmitter receptor/transporter map information and abbreviations, see Supporting Information S2, Table S2. ^#^ *p* < 0.05, uncorrected; *p < 0.05, FDR corrected.

### 3.5 Relationships Between ReHo‐Neurotransmitter Spatial Correlations and Clinical Characteristics

**FIGURE 7:**
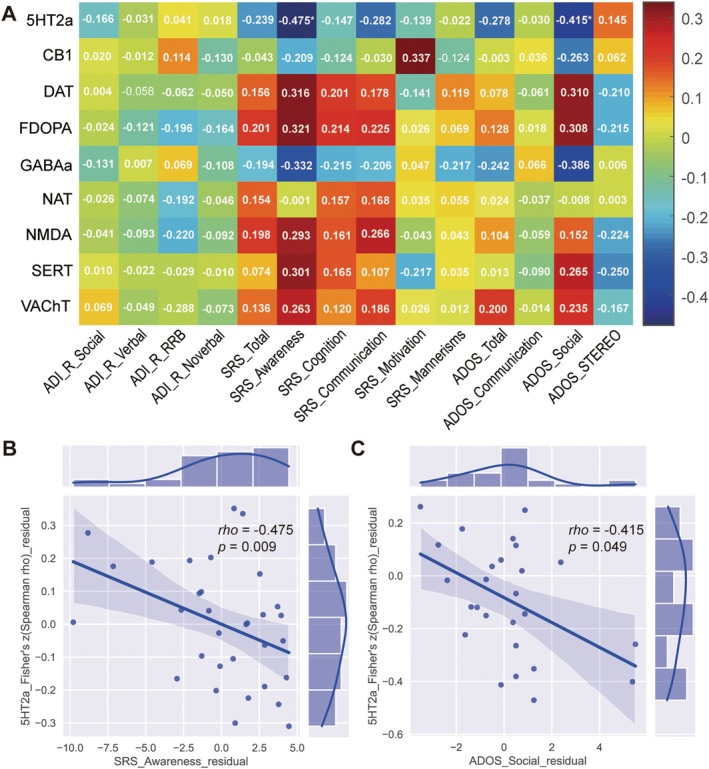
Associations between the ReHo‐neurotransmitter correlation coefficients and the clinical features. (A) Heatmap showing the correlation coefficients between the ReHo‐neurotransmitter correlation coefficients and the clinical features. The colorbar represents the correlation coefficient values. Scatter plots showing that the SRS awareness (B) and ADOS social (C) scores were negatively correlated with the strength of ReHo co‐localization (i.e., Fisher's z‐transformed Spearman correlation coefficient) of the 5HT2a receptor. For details of the neurotransmitter receptor/transporter abbreviations, see Supporting Informations S2, Table S1 and Figure S1. ADI‐R, autism diagnostic interview‐revised; ADOS, autism diagnostic observation schedule; RRB, restricted and repetitive behaviors; SRS, Social Responsiveness Scale; STEREO, stereotyped behaviors.

The relationships between ReHo‐neurotransmitter spatial correlations and the clinical characteristics of patients with ASD were assessed. The SRS awareness (*ρ* = −0.475, *p* = 0.009) and ADOS social (*ρ* = −0.415, *p* = 0.049) scores were negatively correlated with the strength of ReHo colocalization of 5HT2a (Figure 7). No other significant correlations were observed between the clinical features and ReHo‐neurotransmitter spatial correlations.

## 4 Discussion

In this study, the correlations between the spatial distribution of alterations in brain activity and the spatial patterns of specific neurotransmitter receptors/transporters in patients with ASD relative to TDCs were investigated. Patients with ASD exhibited abnormal ReHo in the visuomotor network, cerebro‐cerebellar circuits, angular gyrus, and limbic system. Additionally, ReHo alterations in patients with ASD were significantly co‐localized with the serotonergic, glutamatergic, GABAergic, dopaminergic, noradrenergic, cholinergic, and cannabinoid neurotransmitter systems, including 5HT2a, SERT, NMDA, mGluR5, GABAa, D1, DAT, FDOPA, NAT, VAChT, and CB1, which demonstrated high reproducibility across different neurotransmitter maps. Furthermore, the ReHo colocalization of 5HT2a was negatively correlated with the SRS awareness and ADOS social scores. Our study systematically evaluated multiple specific neurotransmitter system abnormalities in patients with ASD, providing new perspectives for the neurobiological mechanisms underlying ASD as well as a foundation for identifying potential therapeutic targets.

Visuomotor abnormalities are common in patients with ASD [56, 57, 58]. Visual feedback information is sequentially processed through the ventral and dorsal premotor cortices and primary motor cortex to adjust outgoing motor commands [57] and ongoing sensorimotor behavior [56]. Abnormal visual and motor function integration in patients with ASD is associated with impaired social‐communicative skill development [58, 59, 60], early social–emotional deficits [59], and reduced visual feedback sensitivity during motor learning [58, 60]. Our results revealed abnormal activity in the bilateral MOG and right SMA, which are important components of the visual and primary motor cortices, respectively. In addition, motor dysfunction is a core feature of ASD that may limit social interactions and affect social development [12]. Previous studies [12, 56, 61] have reported abnormal brain activity in the SMA of patients with ASD. Cerebellar dysfunction is associated with motor and non‐motor impairments in ASD [62, 63]. Previous studies have indicated that cerebro‐cerebellar circuits are associated with social deficits and repetitive behaviors [64, 65], the core symptoms of ASD, and visuomotor impairments [56, 57]. Abnormalities in right cerebellar crus I and II are associated with more severe ASD impairments [64]. The thalamus is an important node for multiple cerebro‐cerebellar, cortico‐subcortical, and cortico‐cortical circuits, and its dysfunction is closely related to the pathophysiology of ASD [57, 61, 65]. Our results are consistent with those of previous studies.

The angular gyrus plays a crucial role in integrating multisensory information and multiple cognitive processes [66]. Its dysfunction has been implicated in social cognition and social deficits [12, 59, 66]. A large‐scale multimodal neuroimaging meta‐analysis demonstrated reduced spontaneous neural activity in the left angular gyrus [12]. Furthermore, the hippocampus and PHG, as crucial components of the limbic system, are involved in emotional processing, social perception, cognitive function, and working memory in patients with ASD [61, 62, 67]. Hippocampal and PHG dysfunction may lead to poor performance in social cognition and memory tasks [61, 67]. Collectively, dysfunctions of the angular gyrus, hippocampus, and PHG underlie the neural basis of autism‐related symptoms, which is supported by our findings.

Our study also found that ReHo alterations in patients with ASD were significantly colocalized with diverse neurotransmitter systems, with perfect reproducibility across different maps of the same neurotransmitter receptors/transporters (Figures 5 and 6; Tables 2 and 3), indicating the robustness of our methodology and the reproducible nature of our results, consistent with our previous study on Parkinson disease [33].

GABA and glutamate are the primary inhibitory and excitatory neurotransmitters in the brain, respectively, and are essential for brain development and function. Our findings revealed associations between the spatial patterns of alterations in brain activity and the spatial distribution of glutamatergic (NMDA and mGluR5) and GABAergic (GABAa) neurotransmitters in patients with ASD. Numerous animal and human studies have demonstrated that excitatory/inhibitory neurotransmitter imbalance is an important part of the pathogenesis of ASD [3, 11, 68, 69, 70], and that GABA agonists and glutamatergic antagonists improve autistic symptoms [71, 72, 73]. NMDAs are glutamate‐gated ion channels that play a crucial role in brain development and plasticity and modulate the GABAergic system [68]. The association between NMDA function and ASD symptoms has been demonstrated in patients with ASD and in animal models [68, 71, 73, 74]. mGluR5, a member of the group I metabotropic glutamate receptor family, primarily regulates neurogenesis, synaptogenesis, motor functions, and social behavior. It interacts with NMDA and is associated with ASD etiology [71, 73, 75, 76]. GABAa is a ligand‐gated ion channel that inhibits neural excitability and is important for synaptic plasticity, neurogenesis, and cognitive function [77]. Dysfunction of GABAa signaling may contribute to postsynaptic neuronal excitability and abnormal glutamate release [73], which has been implicated in ASD [72, 73, 77]. Previous studies reported that GABA release and GABAa expression are reduced in mouse models of autism [69, 70, 72]. Additionally, GABAa agonists partially reverse autistic‐like behaviors in model mice [72], and clinical trials have confirmed GABAa as an ASD therapeutic target [73, 78]. Thus, targeting the GABA/glutamate pathway is a promising therapeutic target for ASD.

Dysregulation of the serotonin (5‐hydroxytryptamine, 5HT) system is associated with ASD [73, 79, 80, 81]. Hyperserotonemia is the first indicator of ASD, with reductions observed in both 5‐HT receptor and SERT densities across various brain regions in animal models and patients with ASD [27, 73, 80]. A growing body of evidence has confirmed the use of drugs targeting 5HT receptors and SERT agonists/antagonists to alleviate ASD symptoms [73, 79, 81]. In our primary analysis, we found that ReHo alterations were associated with the spatial distribution of 5HT2a and SERT; however, no significant correlations were found for 5HT1a, 5HT1b, and 5HT4. Drugs targeting 5HT2a and SERT have shown promise in improving ASD‐related behavior and cognition [73, 79, 81]. We found that the SRS awareness (*ρ* = −0.475, *p* = 0.009) and ADOS social (*ρ* = −0.415, *p* = 0.049) scores were negatively correlated with the strength of ReHo co‐localization of 5HT2a (Figure 7). These results provide further evidence for the association between 5HT2a and ASD symptoms. Our results support these findings. Although 5HT1a and 5HT1b have been identified as potential therapeutic targets for ASD [73, 79], the results have been inconsistent and contradictory [73, 81]. In both our primary and validation analyses, we observed no significant spatial association between ReHo alterations in patients with ASD and 5HT1b distribution (map: 5HT1b_az_hc36_beliveau; both *ρ* = −0.0455, *p* = 0.0593/0.0631; Tables 2 and 3). However, in the validation analysis, a significant association trend was found (map: 5HT1b_P943_HC22; *ρ* = −0.0851, *p* = 0.0365; Table 3), but the significance disappeared after correction for multiple comparison corrections. The potential of 5HT1a and 5HT1b as therapeutic targets in ASD requires further validation through additional studies. 5HT4 receptors are post‐synaptic receptors involved in cognition and mood. They have been identified as potential therapeutic targets for depression [82, 83] and cognitive impairment [84]; however, few studies have explored the association between 5HT4 expression and ASD. The gut‐brain‐microbiome axis has been implicated in ASD, and serotonin plays a crucial role in the gut‐brain axis [85]. A previous study reported that 5HT4 agonists could improve Al6‐related gastrointestinal function in SERT Ala56 mice [86]. However, further studies are required to ascertain whether these agonists can correct brain and behavioral abnormalities. Additionally, human experiments are necessary to verify the safety and efficacy [86, 87].

Dopamine is a neurotransmitter associated with motor function, motivation, attention, and reward processing [88, 89]. Dysregulation of the dopaminergic system may lead to deficits in reward processing, social behavior and social cognition in ASD and may be an important mechanism for its core symptoms, particularly social deficits [8, 23, 27, 89, 90]. A significant reduction in social cognition measures has been reported among rats with a genetic mutation in the D1 receptors [90]. Moreover, in a mouse model of dendritic cytokine 1 deficiency in the nervous system, a D1 receptor agonist improved social deficits and autism‐like behavior [91]. Significant correlations have been reported between D1 binding and the Autism Spectrum Quotient “attention to detail” subscale score as well as the social acuity score in patients with ASD [23]. Our findings are consistent with these studies, supporting the potential of the D1 receptor as a therapeutic target for ASD. D2 receptors regulate dopamine release and some studies have suggested their association with ASD [13, 22]. However, a spatial association between the D2 receptor density and ReHo in patients with ASD was not observed in our study. Patients in these studies [13, 22] were adults with ASD (all ≥ 18 years of age; mean age, 24.8 and 24.9 years, respectively), whereas the patients in our study were school‐age children (8–13 years of age; mean = 10.4 years). Age differences may have contributed to these inconsistent results. However, further validation is required. FDOPA, an analog of levodopa (L‐DOPA), can be used to assess the dopamine synthesis capacity. Previous studies [8, 27] have shown abnormalities in the DAT and FDOPA in patients with ASD, consistent with our findings. These studies further support the idea that drugs targeting dopamine synthesis and transport are potential therapeutic candidates for ASD.

The endocannabinoid system is a crucial signaling regulatory network that plays a core role in neurodevelopment, synaptic plasticity, neurotransmitter balance (e.g., glutamate, GABA, dopamine, and serotonin), emotional regulation, and social behavior, and is important in the etiopathology of ASD [50, 92]. CB1 is a key receptor in the endocannabinoid system. Previous studies have shown that CB1 deficiency causes abnormalities in social behavior and communication during early development and adulthood in mice [92], and that CB1 activation alleviates ASD‐like behaviors [93]. Cannabidiol [50, 53, 94] and cannabidivarin [51, 54] have recently gained increasing attention owing to their non‐toxic properties and are considered potential candidates for ASD treatment. These compounds can negatively and allosterically modulate the CB1 receptor and regulate the excitatory/inhibitory balance in the brain. Recent studies have revealed that cannabidiol [53] and cannabidivarin [54] can modulate the glutamate‐GABA systems in the brain (i.e., excitatory/inhibitory balance systems). Consistent with these findings, we observed substantial spatial co‐localization between alterations in ReHo in patients with ASD and the spatial distribution of CB1. However, as another study [52] failed to demonstrate their therapeutic efficacy, their therapeutic effects in ASD require further validation [53, 54, 94].

Central cholinergic system dysfunction is believed to underlie ASD‐related behavioral symptoms [27, 95]. A previous study indicated that acetylcholinesterase inhibitors can improve ASD‐related symptoms in mice with autism [96] and children with ASD [27]. However, additional evidence is required to confirm the effectiveness of acetylcholinesterase inhibitors in improving ASD outcomes [97]. VAChT, a protein that regulates acetylcholine secretion, modulates cognitive processing [98] and social behavior and memory [95]. A previous study [99] demonstrated that mice deficient in VAChT exhibited deficits in object and social recognition. Nevertheless, few studies on its role in ASD are available, and more confirmatory studies are required. Collectively, the cholinergic system represents a potential therapeutic target for ASD; however, additional studies are required for validation.

The locus coeruleus‐norepinephrine (LC‐NE) system plays a key role in attentional function and aberrant LC‐NE activation may be associated with reduced social attention [49, 100, 101]. NAT is a key transporter involved in noradrenaline neurotransmitter reuptake. A recent study [23] indicated that NAT function underlies the clinical and cognitive features of patients with ASD and facilitates the development of targeted therapeutic strategies against ASD. However, another study [102] found no association between the NAT gene (*SLC6A2*) and ASD diagnosis or behavioral phenotypes. Our results revealed that ReHo alterations in patients with ASD were significantly colocalized with NAT, which requires further validation.

Our study had some limitations. First, rs‐fMRI data were acquired at one site using two MRI scanners from the same manufacturer and model with the same scanning parameters. Although the samples were perfectly matched between the groups (*χ* ^2^ = 0, *p* = 1.00), potential inter‐scanner batch effects cannot be entirely ruled out despite the use of consistent rs‐fMRI acquisition parameters. We employed ComBat harmonization [5, 44, 45, 46] to mitigate potential biases and non‐biological variability induced by differences in the MRI scanners. Inter‐scanner batch effects were small before ComBat harmonization and were reduced after the process; similar results were observed at the whole‐brain and gray matter voxel levels, as well as at the Brainnetome atlas level (Supporting Informations S6, Figure S3). Moreover, the inclusion of the MRI scanner (head coil) as a covariate in the statistical analysis further minimized the influence of this effect. Further studies with larger sample sizes using the same MRI scanner are needed for validation. Second, our data were derived from a single site, and our study had a relatively small sample size, particularly in the ASD group (*n* = 44), which may have affected the generalizability and stability of the results. Additionally, the missing clinical data, specifically, the total and subscale scores for behavioral scales such as ADOS and SRS, were not available for all participants. This factor may have influenced the effect sizes of the analysis of relationships between ReHo‐neurotransmitter spatial correlations and clinical characteristics. Thus, an analysis with more comprehensive clinical data could potentially reveal additional correlations between ReHo‐neurotransmitter co‐localization and clinical features. Therefore, caution should be exercised when interpreting the findings of this study. Future studies with larger samples, more comprehensive clinical data, and multicenter data are needed to further validate our results. Third, although our study identified statistically significant correlations between ReHo alterations and neurotransmitter maps, the effect sizes were relatively small (Spearman's |*ρ*| mostly < 0.2). These findings are consistent with those of large‐sample studies (*n* > 150 per group) [34, 37, 38], suggesting that this phenomenon may reflect the inherent properties of the spatial associations between brain activity and neurotransmitter distributions. Therefore, caution should be exercised when interpreting these results. Furthermore, our study focused on analyzing the associations between alterations in brain activity patterns and neurotransmitter systems in patients with ASD. It did not investigate the causal relationships between them, nor did it elucidate how neurotransmitter systems affect brain activity patterns or how these patterns, in turn, affect neurotransmitter systems. Future studies should employ more rigorous experimental designs to investigate these causal mechanisms. Fourth, patients with ASD exhibited significantly lower full‐scale and verbal IQ scores than TDCs. Although all adolescent participants (age 8–13 years) were age‐ and sex‐matched, and statistical analyses were adjusted for the confounding effects of age, sex, and head coil, the potential confounding influence of IQ was not well controlled. Future studies should incorporate IQ as a covariate to validate the robustness of our findings. Finally, this was a cross‐sectional study. Future studies should use longitudinal data to assess changes in the spatial associations between neurotransmitters and brain activity over time in patients with ASD and determine how these spatial associations are related to ASD symptoms.

## 5 Conclusions

In conclusion, our study revealed specific brain activity abnormalities in patients with ASD, including those in the visuomotor network, cerebro‐cerebellar circuits, angular gyrus, and limbic system. Furthermore, we comprehensively analyzed the spatial associations between abnormal brain activity patterns in patients with ASD and the density distributions of multiple specific neurotransmitters. These spatial patterns were significantly co‐localized with the serotonergic, glutamatergic, GABAergic, dopaminergic, noradrenergic, cholinergic, and cannabinoid neurotransmitter systems. These findings can enhance our understanding of the mechanisms underlying ASD and may provide potential therapeutic targets for ASD.

## Author Contributions

Study conception, design, data curation, statistical analysis, manuscript preparation, manuscript editing and manuscript review: Dafa Shi; Study conception, design, statistical analysis and manuscript editing: Jitian Guan; data curation and statistical analysis: Guangsong Wang, Shuohua Wu, Caiyu Zhuang, Yumeng Mao, Yanlong Jia, and Nannan Zhao; study conception, design, manuscript editing, manuscript review and funding acquisition: Gen Yan and Renhua Wu. All authors have read and agreed to the published version of the manuscript.

## Funding

This work was supported by the Key Programme of the National Natural Science Foundation of China (grant number 82020108016) and the Science and Technology Project of Xiamen Medical College (grant number K2023‐09).

## Ethics Statement

We included data from a publicly available database. Ethical approval was obtained from the Institutional Review Boards of Kennedy Krieger Institute (data‐sharing institution) and complied with the Declaration of Helsinki. Written informed consent was acquired from all participants or their parents/guardians.

## Consent

All authors have read the manuscript and provided consent for publication.

## Conflicts of Interest

The authors declare no conflicts of interest.

## Supporting information


**Table S1:** Neurotransmitter receptor/transporter density maps considered in our study.
**Figure S1:** Spatial distribution of neurotransmitter receptor/transporter maps included in our study.
**Table S2:** Neurotransmitter density maps considered in validation analysis.
**Table S3:** The brain region information of the Brainnetome 246 atlas.
**Figure S2:** Parcellation scheme of the Brainnetome 246 atlas.
**Table S4:** Brain regions with significant ReHo differences between patients with ASD and TDCs.
**Figure S3:** Principal component analysis scatter plots show that the first two principal components of the whole brain and gray matter voxel levels and Brainnetome atlas based ReHo were visualized in a two‐dimensional scatter plot before and after using ComBat.

## Data Availability

Raw data can be available from the open‐access Autism Brain Imaging Data Exchange (ABIDE) repository, https://fcon_1000.projects.nitrc.org/indi/abide. All data generated or analyzed during this study is included in this published article and its Supporting Information files.
