# Supplementary material for: Mapping Resting‐State Brain Functional Specialization to Neurotransmitter Profiles in Autism Spectrum Disorder
Source: CNS Neurosci Ther. 2025 Nov 24;31(11):e70666. doi: 10.1111/cns.70666 (PMC12641454; doi:10.1111/cns.70666)
Supplement: Supplementary file 1 — Table S1: Neurotransmitter receptor/transporter density maps considered in our study. Figure S1: Spatial distribution of neurotransmitter receptor/transporter maps included in our study. Table S2: Neurotransmitter density maps considered in validation analysis. Table S3: The brain region information of the Brainnetome 246 atlas. Figure S2: Parcellation scheme of the Brainnetome 246 atlas. Table S4: Brain regions with significant ReHo differences between patients with ASD and TDCs. Figure S3: Principal component analysis scatter plots show that the first two principal components of the whole brain and gray matter voxel levels and Brainnetome atlas based ReHo were visualized in a two‐dimensional scatter plot before and after using ComBat. [file CNS-31-e70666-s001.docx]

Supplementary Information for

**Mapping resting-state brain functional specialization to neurotransmitter profiles in autism spectrum disorder**

Dafa Shi^1*^, Jitian Guan^1^, Guangsong Wang^2^, Shuohua Wu^1^, Caiyu Zhuang^1^, Yumeng Mao^1^, Yanlong Jia^3^, Nannan Zhao^4^, Gen Yan^5*^, Renhua Wu^1*^

1. Department of Radiology, The Second Affiliated Hospital of Shantou University Medical College, Shantou, China
2. Department of Radiology, Xiang’an Hospital of Xiamen University, School of Medicine, Xiamen University, Xiamen, China
3. Department of Radiology, Xiangyang Central Hospital, Affiliated Hospital of Hubei University of Arts and Science, Xiangyang, China
4. Center of Morphological Experiment，Medical College of Yanbian University, Yanji, China
5. Department of Radiology, The Second Affiliated Hospital of Xiamen Medical College, Xiamen, China

**Corresponding author**:

Dafa Shi

Department of Radiology, The Second Affiliated Hospital of Shantou University Medical College, Shantou, China

E-mail: [dfshi@stu.edu.cn](mailto:dfshi@stu.edu.cn)

Gen Yan

Department of Radiology, The Second Affiliated Hospital of Xiamen Medical College, Xiamen, China

E-mail: [gyan@stu.edu.cn](mailto:gyan@stu.edu.cn)

Renhua Wu

Department of Radiology, The Second Affiliated Hospital of Shantou University Medical College, Shantou, China

E-mail: [rhwu@stu.edu.cn](mailto:rhwu@stu.edu.cn)

**S1. Neuroimaging acquisition and preprocessing, ReHo calculation**

The rs-fMRI and high resolution T1-weighted structural MRI data were acquired using two 3 Tesla Philips scanners (Achieva; Philips Healthcare, Best, The Netherlands) with an eight-channel or a 32-channel phased array head coil. The ABIDE I data were acquired using an 8-channel head coil, while ABIDE II data were acquired using an 8- or 32-channel head coil. For data collected using the two scanners, rs-fMRI had the same scanning parameters, while 3D T1-weighted anatomical data had similar scanning parameters. The high-resolution three-dimensional T1-weighted structural images were acquired using MPRAGE sequences: (1) eight-channel scanner: coronal slices = 200, repetition time (TR) /echo time (TE) = 8.0/3.7 ms, field of view (FOV) = 256 mm × 200 mm, slice thickness = 1.0 mm, matrix = 256 × 200, voxel size = 1.0 × 1.0 × 1.0 mm^3^, and flip angle (FA) = 8°; (2) 32-channel scanner: axial slices = 150, TR/TE = 8.2/3.7 ms, FOV = 212 mm × 172 mm, slice thickness = 1.0 mm, matrix = 224 × 180, voxel size = 0.95 × 0.96 × 1.0 mm^3^, FA = 8°. Rs-fMRI images were acquired with an echo-planar imaging (EPI) pulse sequences: 156 volumes, slices = 47, TR/TE = 2,500/30 ms, FOV = 256 mm × 256 mm, slice thickness = 3.0 mm, no gap, matrix = 96 × 96, voxel size = 2.67 × 2.67 × 3.00 mm^3^, FA = 75°.

The ReHo analysis was performed using the Data Processing & Analysis for (Resting-State) Brain Imaging (DPABI) toolbox [1]. The preprocessing steps were as follows: (1) the first 8 volumes (20 s) of each participant were removed to ensure a steady-state condition; (2) slice timing correction was used to correct the intra-volume acquisition time delay; (3) image realignment was carried out for head motion correction, we excluded the participants with maximum translation greater than 3.0 mm or maximum rotation greater than 3.0° in our study; (4) the each participant’s high-resolution T1-weighted structural images were co-registered to their rs-fMRI images; (5) The T1 structural images were segmented into gray matter (GM), white matter (WM), and cerebrospinal fluid (CSF); (6) functional data were spatially normalized to the Montreal Neurological Institute (MNI) space and resampled to 3 × 3 × 3 mm³ voxels; (7) the WM, CSF and global signal, 24 head motion parameters, and linear drift were removed as nuisance covariates by a multiple linear regression analysis; (8) bandpass filtering (0.01–0.08 Hz) was used to reduce the effects of low-frequency drift and high-frequency physiological noise. Individual ReHo mapping was performed using a voxel-wise approach by calculating Kendall’s coefficient of concordance for each voxel's time series with its 26 nearest-neighboring voxels. Next, the ReHo value of each voxel was transformed into a z-score for standardization to reduce the influence of individual variability [2, 3]. Finally, the ReHo maps were spatially smoothed using a 4-mm full-width at half-maximum isotropic Gaussian kernel.

**S2. Neurotransmitter receptor/transporter density maps**

**Table S1** Neurotransmitter receptor/transporter density maps considered in our study

| **Receptor/**  **transporter** | **Neurotransmitter** | **Map** | **Reference** |
| --- | --- | --- | --- |
| 5HT1a | Serotonin | 5HT1a_WAY_HC36 | Savli et al.[4] |
| 5HT1b | Serotonin | 5HT1b_az_hc36_beliveau | Beliveau et al.[5] |
| 5HT2a | Serotonin | 5HT2a_cimbi_hc29_beliveau | Beliveau et al.[5]. |
| 5HT4 | Serotonin | 5HT4_sb20_hc59_beliveau | Beliveau et al.[5] |
| CB1 | Cannabinoid | CB1_FMPEPd2_hc22_laurikainen | Hansen et al.[6] |
| D1 | Dopamine | D1_SCH23390_c11 | Kaller et al.[7] |
| D2 | Dopamine | D2_fallypride_hc49_jaworska | Hansen et al.[6] |
| DAT | Dopamine | DAT_DATSPECT | Dukart et al.[8] |
| FDOPA | Dopamine | FDOPA_f18 | García-Gómez et al.[9] |
| GABAa | GABA | GABAa_flumazenil_hc16_norgaard | Nørgaard et al.[10] |
| NAT | Noradrenaline | NAT_MRB_c11 | Hesse et al.[11] |
| NMDA | Glutamate | ge179_29hc_galovic2021 | Galovic et al.[12] |
| SERT | Serotonin | SERT_dasb_hc100_beliveau | Beliveau et al.[5] |
| VAChT | Acetylcholine | VAChT_feobv_hc18_aghourian | Hansen et al.[6] |
| mGluR5 | Glutamate | mGluR5_abp_hc73_smart | Hansen et al.[6] |

**Abbreviations:** 5HT1a, serotonin 5-hydroxytryptamine receptor subtype 1a; 5HT1b, serotonin 5-hydroxytryptamine receptor subtype 1b; 5HT2a, serotonin 5-hydroxytryptamine receptor subtype 2a; 5HT4, serotonin 5-hydroxytryptamine receptor subtype 4; CB1, cannabinoid 1 receptor; D1, dopamine D1 receptor; D2, dopamine D2 receptor; DAT, dopamine transporter; FDOPA, ^18^F fluorodopa; GABAa, gamma-aminobutyric acid type a; NAT, noradrenaline transporter; NMDA, N-methyl-D-aspartic acid receptor; SERT, serotonin transporter; VAChT, vesicular acetylcholine transporter; mGluR5, metabotropic glutamate receptor 5.

**
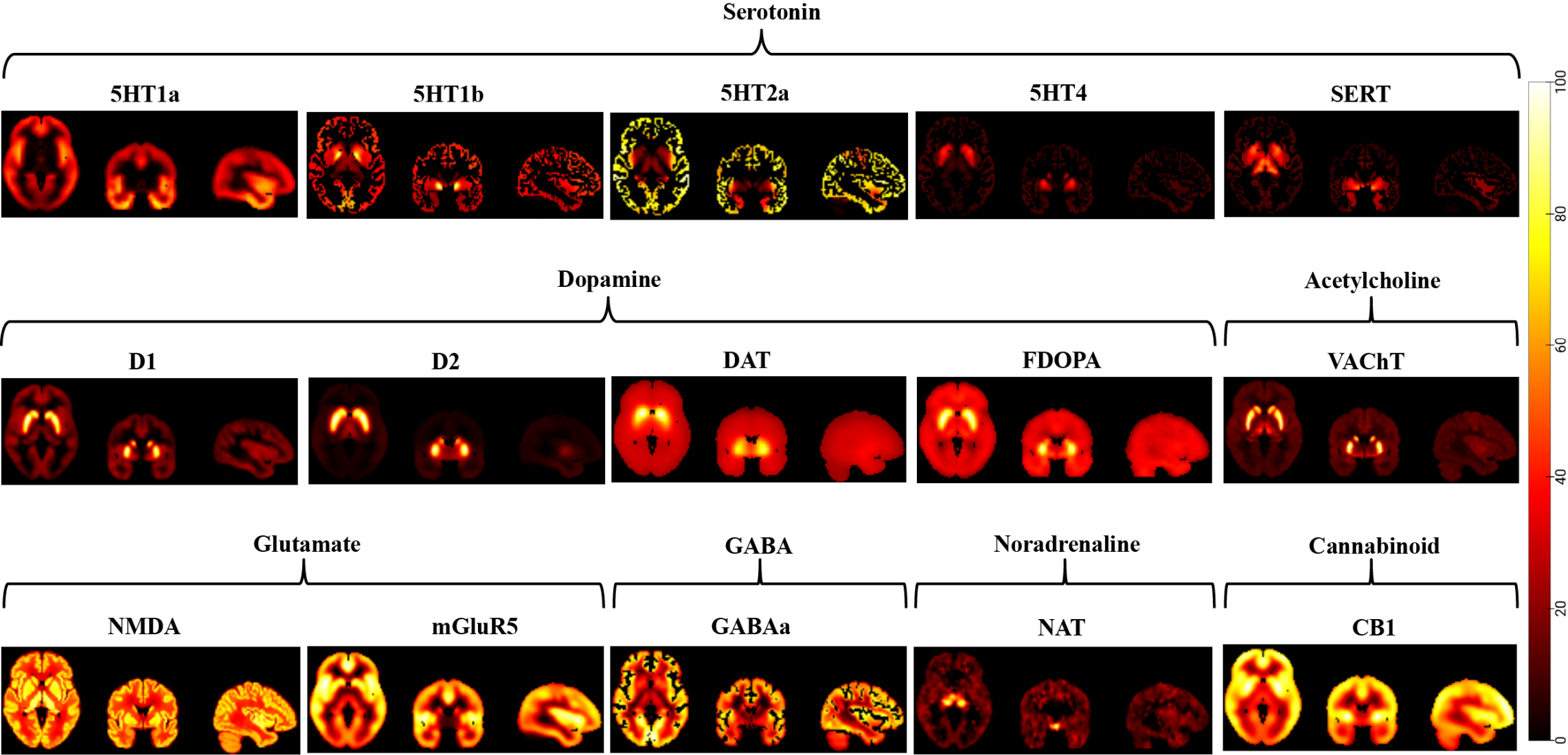
**

**Figure S1** Spatial distribution of neurotransmitter receptor/transporter maps included in our study.

**Abbreviations:** 5HT1a, serotonin 5-hydroxytryptamine receptor subtype 1a; 5HT1b, serotonin 5-hydroxytryptamine receptor subtype 1b; 5HT2a, serotonin 5-hydroxytryptamine receptor subtype 2a; 5HT4, serotonin 5-hydroxytryptamine receptor subtype 4; CB1, cannabinoid 1 receptor; D1, dopamine D1 receptor; D2, dopamine D2 receptor; DAT, dopamine transporter; FDOPA, ^18^F fluorodopa; GABAa, gamma-aminobutyric acid type a; NAT, noradrenaline transporter; NMDA, N-methyl-D-aspartic acid receptor; SERT, serotonin transporter; VAChT, vesicular acetylcholine transporter; mGluR5, metabotropic glutamate receptor 5.

**Table S2** Neurotransmitter density maps considered in validation analysis

| **Receptor/**  **transporter** | **Neurotransmitter** | **Map** | **Volunteers(N)** | **Reference** |
| --- | --- | --- | --- | --- |
| 5HT1a | Serotonin | 5HT1a_WAY_HC36 | 36 | Savli et al.[4] |
|  |  | 5HT1a_cumi_hc8_beliveau | 8 | Hansen et al.[6] |
| 5HT1b | Serotonin | 5HT1b_P943_HC22 | 22 | Savli et al.[4] |
|  |  | 5HT1b_az_hc36_beliveau | 36 | Beliveau et al.[5] |
| 5HT2a | Serotonin | 5HT2a_cimbi_hc29_beliveau | 29 | Beliveau et al.[5]. |
|  |  | 5HT2a_ALT_HC19 | 19 | Savli et al.[4] |
| D2 | Dopamine | D2_fallypride_hc49_jaworska | 49 | Hansen et al.[6] |
|  |  | D2_RACLOPRIDE_c11 | 7 | Alakurtti et al[13] |
| GABAa | GABA | GABAa_flumazenil_hc16_norgaard | 16 | Nørgaard et al.[10] |
|  |  | GABAa_FLUMAZENIL_c11 | 6 | Dukart et al.[8] |
| mGluR5 | Glutamate | mGluR5_abp_hc73_smart | 73 | Hansen et al.[6] |
|  |  | mGluR5_abp_hc28_dubois | 28 | Hansen et al.[6] |
|  |  | mGluR5_abp_hc22_rosaneto | 22 | Hansen et al.[6] |
| SERT | Serotonin | SERT_dasb_hc100_beliveau | 100 | Beliveau et al.[5] |
|  |  | SERT_DASB_HC30 | 30 | Savli et al.[4] |
|  |  | SERT_MADAM_c11 | 10 | Patrik et al. [14] [14] |
| VAChT | Acetylcholine | VAChT_feobv_hc18_aghourian | 18 | Hansen et al.[6] |
|  |  | VAChT_feobv_hc5_bedard | 5 | Hansen et al.[6] |
|  |  | VAChT_feobv_hc4_tuominen | 4 | Hansen et al.[6] |

**Abbreviations:** 5HT1a, serotonin 5-hydroxytryptamine receptor subtype 1a; 5HT1b, serotonin 5-hydroxytryptamine receptor subtype 1b; 5HT2a, serotonin 5-hydroxytryptamine receptor subtype 2a; D2, dopamine D2 receptor; GABAa, gamma-aminobutyric acid type a; mGluR5, metabotropic glutamate receptor 5; SERT, serotonin transporter; VAChT, vesicular acetylcholine transporter.

**S3. The brain region information of the Brainnetome 246 atlas**

**Table S3** The brain region information of the Brainnetome 246 atlas

| **Lobe** | **Gyrus** | **Left and Right Hemisphere** | **Label ID.L** | **Label ID.R** | **lh.MNI(X,Y,Z)** | **rh.MNI(X,Y,Z)** |
| --- | --- | --- | --- | --- | --- | --- |
| **Frontal Lobe** | SFG, Superior Frontal Gyrus | SFG_L(R)_7_1 | 1 | 2 | -5 ,15, 54 | 7, 16, 54 |
|  |  | SFG_L(R)_7_2 | 3 | 4 | -18, 24, 53 | 22, 26, 51 |
|  |  | SFG_L(R)_7_3 | 5 | 6 | -11, 49, 40 | 13, 48, 40 |
|  |  | SFG_L(R)_7_4 | 7 | 8 | -18, -1, 65 | 20, 4, 64 |
|  |  | SFG_L(R)_7_5 | 9 | 10 | -6, -5, 58 | 7, -4, 60 |
|  |  | SFG_L(R)_7_6 | 11 | 12 | -5, 36, 38 | 6, 38, 35 |
|  |  | SFG_L(R)_7_7 | 13 | 14 | -8, 56, 15 | 8, 58, 13 |
|  | MFG, Middle Frontal Gyrus | MFG_L(R)_7_1 | 15 | 16 | -27, 43, 31 | 30, 37, 36 |
|  |  | MFG_L(R)_7_2 | 17 | 18 | -42, 13, 36 | 42, 11, 39 |
|  |  | MFG_L(R)_7_3 | 19 | 20 | -28, 56, 12 | 28, 55, 17 |
|  |  | MFG_L(R)_7_4 | 21 | 22 | -41, 41, 16 | 42, 44, 14 |
|  |  | MFG_L(R)_7_5 | 23 | 24 | -33, 23, 45 | 42, 27, 39 |
|  |  | MFG_L(R)_7_6 | 25 | 26 | -32, 4, 55 | 34, 8, 54 |
|  |  | MFG_L(R)_7_7 | 27 | 28 | -26, 60, -6 | 25, 61, -4 |
|  | IFG, Inferior Frontal Gyrus | IFG_L(R)_6_1 | 29 | 30 | -46, 13, 24 | 45, 16, 25 |
|  |  | IFG_L(R)_6_2 | 31 | 32 | -47, 32, 14 | 48, 35, 13 |
|  |  | IFG_L(R)_6_3 | 33 | 34 | -53, 23, 11 | 54, 24, 12 |
|  |  | IFG_L(R)_6_4 | 35 | 36 | -49, 36, -3 | 51, 36, -1 |
|  |  | IFG_L(R)_6_5 | 37 | 38 | -39, 23, 4 | 42, 22, 3 |
|  |  | IFG_L(R)_6_6 | 39 | 40 | -52, 13, 6 | 54, 14, 11 |
|  | OrG, Orbital Gyrus | OrG_L(R)_6_1 | 41 | 42 | -7, 54, -7 | 6, 47, -7 |
|  |  | OrG_L(R)_6_2 | 43 | 44 | -36, 33, -16 | 40, 39, -14 |
|  |  | OrG_L(R)_6_3 | 45 | 46 | -23, 38, -18 | 23, 36, -18 |
|  |  | OrG_L(R)_6_4 | 47 | 48 | -6, 52, -19 | 6, 57, -16 |
|  |  | OrG_L(R)_6_5 | 49 | 50 | -10, 18, -19 | 9, 20, -19 |
|  |  | OrG_L(R)_6_6 | 51 | 52 | -41, 32, -9 | 42, 31, -9 |
|  | PrG, Precentral Gyrus | PrG_L(R)_6_1 | 53 | 54 | -49, -8, 39 | 55, -2, 33 |
|  |  | PrG_L(R)_6_2 | 55 | 56 | -32, -9, 58 | 33, -7, 57 |
|  |  | PrG_L(R)_6_3 | 57 | 58 | -26, -25, 63 | 34, -19, 59 |
|  |  | PrG_L(R)_6_4 | 59 | 60 | -13, -20, 73 | 15, -22, 71 |
|  |  | PrG_L(R)_6_5 | 61 | 62 | -52, 0, 8 | 54, 4, 9 |
|  |  | PrG_L(R)_6_6 | 63 | 64 | -49, 5, 30 | 51, 7, 30 |
|  | PCL, Paracentral Lobule | PCL_L(R)_2_1 | 65 | 66 | -8, -38, 58 | 10, -34, 54 |
|  |  | PCL_L(R)_2_2 | 67 | 68 | -4, -23, 61 | 5, -21, 61 |
| **Temporal Lobe** | STG, Superior Temporal Gyrus | STG_L(R)_6_1 | 69 | 70 | -32, 14, -34 | 31, 15, -34 |
|  |  | STG_L(R)_6_2 | 71 | 72 | -54, -32, 12 | 54, -24, 11 |
|  |  | STG_L(R)_6_3 | 73 | 74 | -50, -11, 1 | 51, -4, -1 |
|  |  | STG_L(R)_6_4 | 75 | 76 | -62, -33, 7 | 66, -20, 6 |
|  |  | STG_L(R)_6_5 | 77 | 78 | -45, 11, -20 | 47, 12, -20 |
|  |  | STG_L(R)_6_6 | 79 | 80 | -55, -3, -10 | 56, -12, -5 |
|  | MTG, Middle Temporal Gyrus | MTG_L(R)_4_1 | 81 | 82 | -65, -30, -12 | 65, -29, -13 |
|  |  | MTG_L(R)_4_2 | 83 | 84 | -53, 2, -30 | 51, 6, -32 |
|  |  | MTG_L(R)_4_3 | 85 | 86 | -59, -58, 4 | 60, -53, 3 |
|  |  | MTG_L(R)_4_4 | 87 | 88 | -58, -20, -9 | 58, -16, -10 |
|  | ITG, Inferior Temporal Gyrus | ITG_L(R)_7_1 | 89 | 90 | -45, -26, -27 | 46, -14, -33 |
|  |  | ITG_L(R)_7_2 | 91 | 92 | -51, -57, -15 | 53, -52, -18 |
|  |  | ITG_L(R)_7_3 | 93 | 94 | -43, -2, -41 | 40, 0, -43 |
|  |  | ITG_L(R)_7_4 | 95 | 96 | -56, -16, -28 | 55, -11, -32 |
|  |  | ITG_L(R)_7_5 | 97 | 98 | -55, -60, -6 | 54, -57, -8 |
|  |  | ITG_L(R)_7_6 | 99 | 100 | -59, -42, -16 | 61, -40, -17 |
|  |  | ITG_L(R)_7_7 | 101 | 102 | -55, -31, -27 | 54, -31, -26 |
|  | FuG, Fusiform Gyrus | FuG_L(R)_3_1 | 103 | 104 | -33, -16, -32 | 33, -15, -34 |
|  |  | FuG_L(R)_3_2 | 105 | 106 | -31, -64, -14 | 31, -62, -14 |
|  |  | FuG_L(R)_3_3 | 107 | 108 | -42, -51, -17 | 43, -49, -19 |
|  | PhG, Parahippocampal Gyrus | PhG_L(R)_6_1 | 109 | 110 | -27, -7, -34 | 28, -8, -33 |
|  |  | PhG_L(R)_6_2 | 111 | 112 | -25, -25, -26 | 26, -23, -27 |
|  |  | PhG_L(R)_6_3 | 113 | 114 | -28, -32, -18 | 30, -30, -18 |
|  |  | PhG_L(R)_6_4 | 115 | 116 | -19, -12, -30 | 19, -10, -30 |
|  |  | PhG_L(R)_6_5 | 117 | 118 | -23, 2, -32 | 22, 1, -36 |
|  |  | PhG_L(R)_6_6 | 119 | 120 | -17, -39, -10 | 19, -36, -11 |
|  | pSTS, posterior Superior Temporal Sulcus | pSTS_L(R)_2_1 | 121 | 122 | -54, -40, 4 | 53, -37, 3 |
|  |  | pSTS_L(R)_2_2 | 123 | 124 | -52, -50, 11 | 57, -40, 12 |
| **Parietal Lobe** | SPL, Superior Parietal Lobule | SPL_L(R)_5_1 | 125 | 126 | -16, -60, 63 | 19, -57, 65 |
|  |  | SPL_L(R)_5_2 | 127 | 128 | -15, -71, 52 | 19, -69, 54 |
|  |  | SPL_L(R)_5_3 | 129 | 130 | -33, -47, 50 | 35, -42, 54 |
|  |  | SPL_L(R)_5_4 | 131 | 132 | -22, -47, 65 | 23, -43, 67 |
|  |  | SPL_L(R)_5_5 | 133 | 134 | -27, -59, 54 | 31, -54, 53 |
|  | IPL, Inferior Parietal Lobule | IPL_L(R)_6_1 | 135 | 136 | -34, -80, 29 | 45, -71, 20 |
|  |  | IPL_L(R)_6_2 | 137 | 138 | -38, -61, 46 | 39, -65, 44 |
|  |  | IPL_L(R)_6_3 | 139 | 140 | -51, -33, 42 | 47, -35, 45 |
|  |  | IPL_L(R)_6_4 | 141 | 142 | -56, -49, 38 | 57, -44, 38 |
|  |  | IPL_L(R)_6_5 | 143 | 144 | -47, -65, 26 | 53, -54, 25 |
|  |  | IPL_L(R)_6_6 | 145 | 146 | -53, -31, 23 | 55, -26, 26 |
|  | Pcun, Precuneus | PCun_L(R)_4_1 | 147 | 148 | -5, -63, 51 | 6, -65, 51 |
|  |  | PCun_L(R)_4_2 | 149 | 150 | -8, -47, 57 | 7, -47, 58 |
|  |  | PCun_L(R)_4_3 | 151 | 152 | -12, -67, 25 | 16, -64, 25 |
|  |  | PCun_L(R)_4_4 | 153 | 154 | -6, -55, 34 | 6, -54, 35 |
|  | PoG, Postcentral Gyrus | PoG_L(R)_4_1 | 155 | 156 | -50, -16, 43 | 50, -14, 44 |
|  |  | PoG_L(R)_4_2 | 157 | 158 | -56, -14, 16 | 56, -10, 15 |
|  |  | PoG_L(R)_4_3 | 159 | 160 | -46, -30, 50 | 48, -24, 48 |
|  |  | PoG_L(R)_4_4 | 161 | 162 | -21, -35, 68 | 20, -33, 69 |
| **Insular Lobe** | INS, Insular Gyrus | INS_L(R)_6_1 | 163 | 164 | -36, -20, 10 | 37, -18, 8 |
|  |  | INS_L(R)_6_2 | 165 | 166 | -32, 14, -13 | 33, 14, -13 |
|  |  | INS_L(R)_6_3 | 167 | 168 | -34, 18, 1 | 36, 18, 1 |
|  |  | INS_L(R)_6_4 | 169 | 170 | -38, -4, -9 | 39, -2, -9 |
|  |  | INS_L(R)_6_5 | 171 | 172 | -38, -8, 8 | 39, -7, 8 |
|  |  | INS_L(R)_6_6 | 173 | 174 | -38, 5, 5 | 38, 5, 5 |
| **Limbic Lobe** | CG, Cingulate Gyrus | CG_L(R)_7_1 | 175 | 176 | -4, -39, 31 | 4, -37, 32 |
|  |  | CG_L(R)_7_2 | 177 | 178 | -3, 8, 25 | 5, 22, 12 |
|  |  | CG_L(R)_7_3 | 179 | 180 | -6, 34, 21 | 5, 28, 27 |
|  |  | CG_L(R)_7_4 | 181 | 182 | -8, -47, 10 | 9, -44, 11 |
|  |  | CG_L(R)_7_5 | 183 | 184 | -5, 7, 37 | 4, 6, 38 |
|  |  | CG_L(R)_7_6 | 185 | 186 | -7, -23, 41 | 6, -20, 40 |
|  |  | CG_L(R)_7_7 | 187 | 188 | -4, 39, -2 | 5, 41, 6 |
| **Occipital Lobe** | MVOcC*,* MedioVentral Occipital Cortex | MVOcC _L(R)_5_1 | 189 | 190 | -11, -82, -11 | 10, -85, -9 |
|  |  | MVOcC _L(R)_5_2 | 191 | 192 | -5, -81, 10 | 7, -76, 11 |
|  |  | MVOcC _L(R)_5_3 | 193 | 194 | -6, -94, 1 | 8, -90, 12 |
|  |  | MVOcC _L(R)_5_4 | 195 | 196 | -17, -60, -6 | 18, -60, -7 |
|  |  | MVOcC _L(R)_5_5 | 197 | 198 | -13, -68, 12 | 15, -63, 12 |
|  | LOcC, lateral Occipital Cortex | LOcC_L(R)_4_1 | 199 | 200 | -31, -89, 11 | 34, -86, 11 |
|  |  | LOcC _L(R)_4_2 | 201 | 202 | -46, -74, 3 | 48, -70, -1 |
|  |  | LOcC _L(R)_4_3 | 203 | 204 | -18, -99, 2 | 22, -97, 4 |
|  |  | LOcC_L(R)_4_4 | 205 | 206 | -30, -88, -12 | 32, -85, -12 |
|  |  | LOcC _L(R)_2_1 | 207 | 208 | -11, -88, 31 | 16, -85, 34 |
|  |  | LOcC _L(R)_2_2 | 209 | 210 | -22, -77, 36 | 29, -75, 36 |
| **Subcortical Nuclei** | Amyg, Amygdala | Amyg_L(R)_2_1 | 211 | 212 | -19, -2, -20 | 19, -2, -19 |
|  |  | Amyg_L(R)_2_2 | 213 | 214 | -27, -4, -20 | 28, -3, -20 |
|  | Hipp, Hippocampus | Hipp_L(R)_2_1 | 215 | 216 | -22, -14, -19 | 22, -12, -20 |
|  |  | Hipp_L(R)_2_2 | 217 | 218 | -28, -30, -10 | 29, -27, -10 |
|  | BG, Basal Ganglia | BG_L(R)_6_1 | 219 | 220 | -12, 14, 0 | 15, 14, -2 |
|  |  | BG_L(R)_6_2 | 221 | 222 | -22, -2, 4 | 22, -2, 3 |
|  |  | BG_L(R)_6_3 | 223 | 224 | -17, 3, -9 | 15, 8, -9 |
|  |  | BG_L(R)_6_4 | 225 | 226 | -23, 7, -4 | 22, 8, -1 |
|  |  | BG_L(R)_6_5 | 227 | 228 | -14, 2, 16 | 14, 5, 14 |
|  |  | BG_L(R)_6_6 | 229 | 230 | -28, -5, 2 | 29, -3, 1 |
|  | Tha, Thalamus | Tha_L(R)_8_1 | 231 | 232 | -7, -12, 5 | 7, -11, 6 |
|  |  | Tha_L(R)_8_2 | 233 | 234 | -18, -13, 3 | 12, -14, 1 |
|  |  | Tha_L(R)_8_3 | 235 | 236 | -18, -23, 4 | 18, -22, 3 |
|  |  | Tha_L(R)_8_4 | 237 | 238 | -7, -14, 7 | 3, -13, 5 |
|  |  | Tha_L(R)_8_5 | 239 | 240 | -16, -24, 6 | 15, -25, 6 |
|  |  | Tha_L(R)_8_6 | 241 | 242 | -15, -28, 4 | 13, -27, 8 |
|  |  | Tha_L(R)_8_7 | 243 | 244 | -12, -22, 13 | 10, -14, 14 |
|  |  | Tha_L(R)_8_8 | 245 | 246 | -11, -14, 2 | 13, -16, 7 |

**
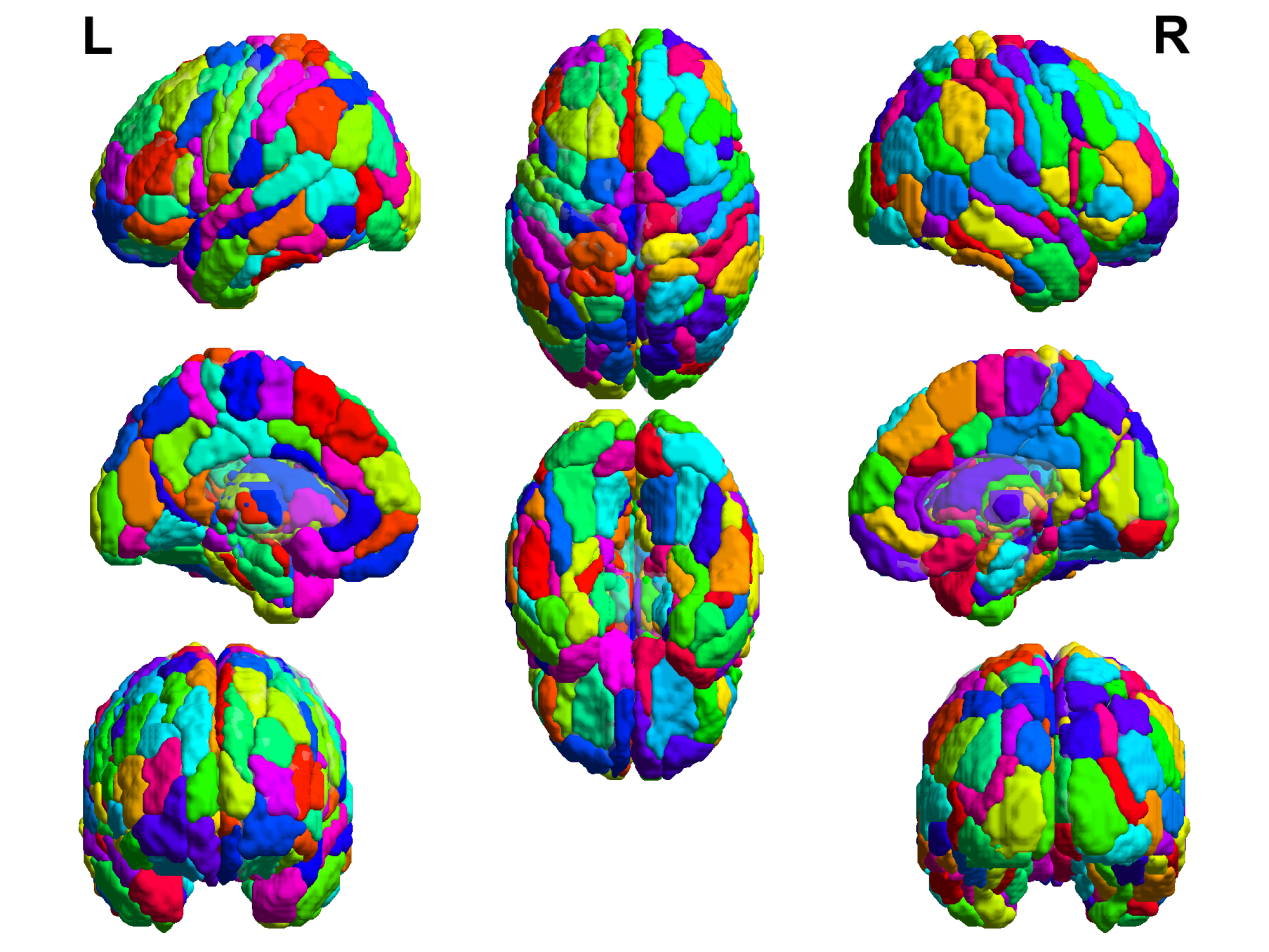
**

**Figure S2** Parcellation scheme of the Brainnetome 246 atlas.

**S4. Spatial correlation analysis between ReHo alterations and** **neurotransmitter receptor/transporter density maps**

The JuSpace toolbox (version 1.5, <https://github.com/juryxy/JuSpace>) [15] was used to evaluate the spatial associations between the spatial patterns of ReHo alterations in patients with ASD relative to TDCs and the specific neurotransmitter receptor/transporter distribution maps of PET/SPECT. Covariates, such as age, sex, and head coil, were adjusted by regression before the spatial correlation analysis [16, 17]. To quantify ReHo alterations in patients with ASD relative to TDCs, we implemented the following computational procedure: First, the group-level mean (Mean_Reho_TDC_) and standard deviation (Std_ReHo_TDC_) of ReHo values were calculated for the TDC cohort. Subsequently, the ReHo map of each patient with ASD was converted to a z-score by subtracting Mean_Reho_TDC_ and dividing by Std_ReHo_TDC_. This process generated individualized ReHo alteration map representing the spatial pattern and magnitude of deviation in each patient with ASD relative to the TDC population. All analyses were performed using the age-, sex-, and head coil-adjusted z-transformed individual ReHo maps. Then, Fisher's z-transformed Spearman’s correlation coefficients were calculated between z-transformed individual ReHo maps of patients with ASD relative to TDCs and the selected neurotransmitter receptor/transporter maps (15 and 19 neurotransmitter maps for the primary and validation analyses, respectively). Calculations were performed using the JuSpace toolbox [15] with computing option 5 and the Brainnetome 246 atlas [18].Specifically, we used the Brainnetome 246 atlas to extract the mean region ReHo values for all patients with ASD and TDCs and the mean neurotransmitter receptor/transporter density values for all selected neurotransmitter receptor/transporter maps for 246 regions. Subsequently, we obtained individual z-transformed maps of list 1 (patients with ASD) relative to list 2 (TDCs) and computed Fisher's z-transformed Spearman correlation coefficients between the ReHo maps (z-transformed) and the spatial distribution of the respective neurotransmitter receptor/transporter density maps. The grey matter probability map was used to adjust for spatial autocorrelation and partial-volume effects. To assess the statistical significance of the observed correlation coefficients, exact permutation-based *p*-values were calculated using 10,000 permutations to determine whether the observed correlation coefficients were statistically different from zero using one-sample t-tests. The false discovery rate (FDR) used to correct for multiple comparisons (the number of neurotransmitter maps: 15 and 19 for the primary and validation analyses, respectively; *p* < 0.05).

**S5. ReHo alterations in patients with ASD**

**Table S4** Brain regions with significant ReHo differences between patients with ASD and TDCs

| Cluster location | Peak MNI coordinate | | | No. of voxels | *t*-value |
| --- | --- | --- | --- | --- | --- |
|  | x | y | z |  |  |
| Right cerebellum crus I and II | 39 | – 75 | – 39 | 142 | – 4.89 |
| Right middle occipital gyrus | 30 | – 81 | 21 | 167 | – 5.34 |
| Left thalamus, parahippocampal gyrus, hippocampus | – 18 | – 33 | – 3 | 69 | 5.57 |
| Left middle occipital gyrus, angular gyrus | – 42 | – 72 | 36 | 112 | – 4.53 |
| Right supplementary motor area | 12 | 0 | 51 | 67 | 4.63 |

**S6. Visualization of the ComBat corrected head coil batch effects**

**
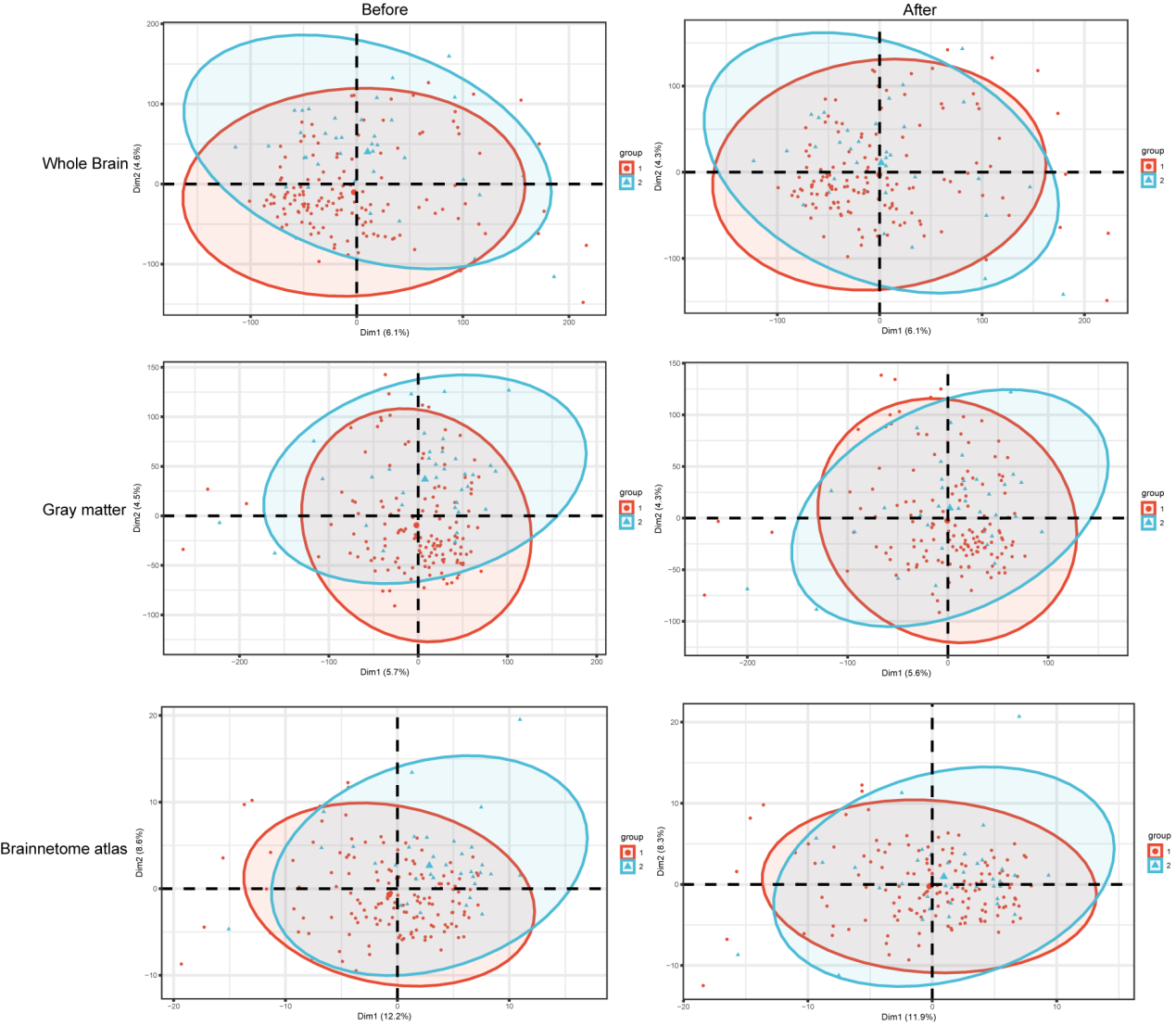
**

**Figure S3** Principal component analysis scatter plots show that the first two principal components of the whole brain and gray matter voxel levels and Brainnetome atlas based ReHo were visualized in a two-dimensional scatter plot before and after using ComBat.

**References**

1. Yan CG, Wang XD, Zuo XN, Zang YF. DPABI: Data Processing & Analysis for (Resting-State) Brain Imaging. *Neuroinformatics* 2016; **14**(3)**:** 339-351.

2. Shi D, Li Y, Zhang H, Yao X, Wang S, Wang G *et al.* Machine Learning of Schizophrenia Detection with Structural and Functional Neuroimaging. *Dis Markers* 2021; **2021:** 9963824.

3. Lei X, Zhong M, Liu Y, Jin X, Zhou Q, Xi C *et al.* A resting-state fMRI study in borderline personality disorder combining amplitude of low frequency fluctuation, regional homogeneity and seed based functional connectivity. *J Affect Disord* 2017; **218:** 299-305.

4. Savli M, Bauer A, Mitterhauser M, Ding YS, Hahn A, Kroll T *et al.* Normative database of the serotonergic system in healthy subjects using multi-tracer PET. *Neuroimage* 2012; **63**(1)**:** 447-459.

5. Beliveau V, Ganz M, Feng L, Ozenne B, Hojgaard L, Fisher PM *et al.* A High-Resolution In Vivo Atlas of the Human Brain's Serotonin System. *J Neurosci* 2017; **37**(1)**:** 120-128.

6. Hansen JY, Shafiei G, Markello RD, Smart K, Cox SML, Norgaard M *et al.* Mapping neurotransmitter systems to the structural and functional organization of the human neocortex. *Nat Neurosci* 2022; **25**(11)**:** 1569-1581.

7. Kaller S, Rullmann M, Patt M, Becker GA, Luthardt J, Girbardt J *et al.* Test-retest measurements of dopamine D(1)-type receptors using simultaneous PET/MRI imaging. *Eur J Nucl Med Mol Imaging* 2017; **44**(6)**:** 1025-1032.

8. Dukart J, Holiga S, Chatham C, Hawkins P, Forsyth A, McMillan R *et al.* Cerebral blood flow predicts differential neurotransmitter activity. *Sci Rep* 2018; **8**(1)**:** 4074.

9. García-Gómez FJ, García-Solís D, Luis-Simón FJ, Marín-Oyaga VA, Carrillo F, Mir P *et al.* Elaboración de una plantilla de SPM para la normalización de imágenes de SPECT con 123I-Ioflupano. *Revista Española de Medicina Nuclear e Imagen Molecular* 2013; **32**(6)**:** 350-356.

10. Norgaard M, Beliveau V, Ganz M, Svarer C, Pinborg LH, Keller SH *et al.* A high-resolution in vivo atlas of the human brain's benzodiazepine binding site of GABA(A) receptors. *Neuroimage* 2021; **232:** 117878.

11. Hesse S, Becker GA, Rullmann M, Bresch A, Luthardt J, Hankir MK *et al.* Central noradrenaline transporter availability in highly obese, non-depressed individuals. *Eur J Nucl Med Mol Imaging* 2017; **44**(6)**:** 1056-1064.

12. Galovic M, Al-Diwani A, Vivekananda U, Torrealdea F, Erlandsson K, Fryer TD *et al.* In vivo NMDA receptor function in people with NMDA receptor antibody encephalitis. *medrxiv* 2021**:** 2021.2012.2004.21267226.

13. Alakurtti K, Johansson JJ, Joutsa J, Laine M, Backman L, Nyberg L *et al.* Long-term test-retest reliability of striatal and extrastriatal dopamine D2/3 receptor binding: study with [(11)C]raclopride and high-resolution PET. *J Cereb Blood Flow Metab* 2015; **35**(7)**:** 1199-1205.

14. https://www.nitrc.org/projects/ki-5htt.

15. Dukart J, Holiga S, Rullmann M, Lanzenberger R, Hawkins PCT, Mehta MA *et al.* JuSpace: A tool for spatial correlation analyses of magnetic resonance imaging data with nuclear imaging derived neurotransmitter maps. *Hum Brain Mapp* 2021; **42**(3)**:** 555-566.

16. Premi E, Pengo M, Mattioli I, Cantoni V, Dukart J, Gasparotti R *et al.* Early neurotransmitters changes in prodromal frontotemporal dementia: A GENFI study. *Neurobiol Dis* 2023; **179:** 106068.

17. Shi D, Wu S, Zhuang C, Mao Y, Wang Q, Zhai H *et al.* Multimodal data fusion reveals functional and neurochemical correlates of Parkinson's disease. *Neurobiol Dis* 2024; **197:** 106527.

18. Fan L, Li H, Zhuo J, Zhang Y, Wang J, Chen L *et al.* The Human Brainnetome Atlas: A New Brain Atlas Based on Connectional Architecture. *Cereb Cortex* 2016; **26**(8)**:** 3508-3526.
